# Fecal microbiota in congenital chloride diarrhea and inflammatory bowel disease

**DOI:** 10.1371/journal.pone.0269561

**Published:** 2022-06-09

**Authors:** Satu Wedenoja, Aki Saarikivi, Jani Mälkönen, Saara Leskinen, Markku Lehto, Krishna Adeshara, Jetta Tuokkola, Anne Nikkonen, Laura Merras-Salmio, Miikka Höyhtyä, Sohvi Hörkkö, Anu Haaramo, Anne Salonen, Willem M. de Vos, Katri Korpela, Kaija-Leena Kolho

**Affiliations:** 1 Obstetrics and Gynecology, University of Helsinki and Helsinki University Hospital, Helsinki, Finland; 2 Stem Cells and Metabolism Research Program, University of Helsinki, and Folkhälsan Research Center, Helsinki, Finland; 3 Children’s Hospital, Pediatric Research Center, University of Helsinki and Helsinki University Hospital, Helsinki, Finland; 4 Department of Pediatrics, University of Kuopio and Kuopio University Hospital, Kuopio, Finland; 5 Folkhälsan Institute of Genetics, Folkhälsan Research Center, Helsinki, Finland; 6 Abdominal Center, Nephrology, University of Helsinki and Helsinki University Hospital, Helsinki, Finland; 7 Clinical and Molecular Metabolism, Faculty of Medicine Research Programs, University of Helsinki, Helsinki, Finland; 8 Faculty of Medicine and Health Technology, Tampere University, Tampere, Finland; 9 Medical Microbiology and Immunology, Research Unit of Biomedicine, University of Oulu, Oulu, Finland; 10 Medical Research Center Oulu, Oulu University Hospital and University of Oulu, Oulu, Finland; 11 Department of Otorhinolaryngology, Helsinki University Hospital, Helsinki, Finland; 12 Human Microbiome Research Program, Faculty of Medicine, University of Helsinki, Helsinki, Finland; 13 Laboratory of Microbiology, Wageningen University, Wageningen, the Netherlands; INRAE, FRANCE

## Abstract

**Background and aims:**

Subjects with congenital chloride diarrhea (CLD; a defect in solute carrier family 26 member 3 (SLC26A3)) are prone to inflammatory bowel disease (IBD). We investigated fecal microbiota in CLD and CLD-associated IBD. We also tested whether microbiota is modulated by supplementation with the short-chain fatty acid butyrate.

**Subjects and methods:**

We recruited 30 patients with CLD for an observational 3-week follow-up study. Thereafter, 16 consented to oral butyrate substitution for a 3-week observational period. Fecal samples, collected once a week, were assayed for calprotectin and potential markers of inflammation, and studied by 16S ribosomal ribonucleic acid (rRNA) gene amplicon sequencing and compared to that of 19 healthy controls and 43 controls with Crohn’s disease. Data on intestinal symptoms, diet and quality of life were collected.

**Results:**

Patients with CLD had increased abundances of Proteobacteria, *Veillonella*, and *Prevotella*, and lower abundances of normally dominant taxa *Ruminococcaceae* and *Lachnospiraceae* when compared with healthy controls and Crohn´s disease. No major differences in fecal microbiota were found between CLD and CLD-associated IBD (including two with yet untreated IBD). Butyrate was poorly tolerated and showed no major effects on fecal microbiota or biomarkers in CLD.

**Conclusions:**

Fecal microbiota in CLD is different from that of healthy subjects or Crohn´s disease. Unexpectedly, no changes in the microbiota or fecal markers characterized CLD-associated IBD, an entity with high frequency among patients with CLD.

## Introduction

A rare autosomal recessive disease congenital chloride diarrhea (CLD; OMIM #214700) is caused by mutations in the *solute carrier family 26 member 3* (*SLC26A3* alias *DRA*) gene on chromosome 7q22.3–31.1 [1, 2]. *SLC26A3* encodes for a major apical epithelial chloride-bicarbonate exchanger of the terminal ileum and colon [3]. Loss of SLC26A3-mediated anion transport in CLD, and the coupled failure of sodium-hydrogen exchanger 3 (NHE3 alias SLC9A3) function, impair intestinal sodium chloride and fluid reabsorption [4–7]. The simple measurement of high fecal chloride (>90 mmol/L) or *SLC26A3* mutation analysis confirm CLD diagnosis [8, 9]. While options to resolve chronic diarrhea are missing, oral salt substitution with sodium and potassium chloride allows favorable outcome in CLD and prevents complications such as chronic volume and electrolyte depletion and kidney disease [10, 11].

The risk for inflammatory bowel disease (IBD) is elevated both in human CLD and in *slc26a3*-deficient mice [7, 12, 13]. Several studies have linked *SLC26A3* polymorphisms or downregulation to ulcerative colitis (UC) [14–16], and low or absent *SLC26A3* expression to intestinal adenomas and adenocarcinomas [17, 18]. Importantly, *SLC26A3* downregulation seems to be a universal phenomenon of intestinal inflammation, and in the absence of any primary genetic defect, is linked to intestinal inflammation induced by various triggers such as chemicals, pathogens, or cytokines [12, 19, 20]. To support the role of altered sodium and chloride transport in human IBD, intestinal inflammation is associated both with CLD and with another rare human disease, congenital sodium diarrhea (CSD) [21–23].

Although dysregulated water and electrolyte transport is a potential driver of IBD [24, 25], SLC26A3 might have broader effects on mucosal health through maintenance of the mucin biosynthesis and intestinal barrier function [26, 27]. Whether gut microbiota is affected in CLD, or plays a role in CLD-associated IBD, remains unknown. We and others previously found that oral administration of short-chain fatty acid (SCFA) butyrate, the most essential end-product of bacterial carbohydrate fermentation in the colon and the principal nutrient for colonocytes [28], reduces the diarrhea in some patients with CLD [29–31]. While these individual responses are poorly understood, luminal butyrate might increase intestinal sodium chloride and fluid reabsorption by modulating colonic epithelial ion transport [28]. Here, we utilized one of the largest founder populations of CLD to study fecal microbiota in patients with CLD and in CLD-associated IBD. We further tested whether the changes in fecal microbiota could be restored by oral SCFA butyrate.

## Patients and methods

### Subjects and controls

**Fig 1:**
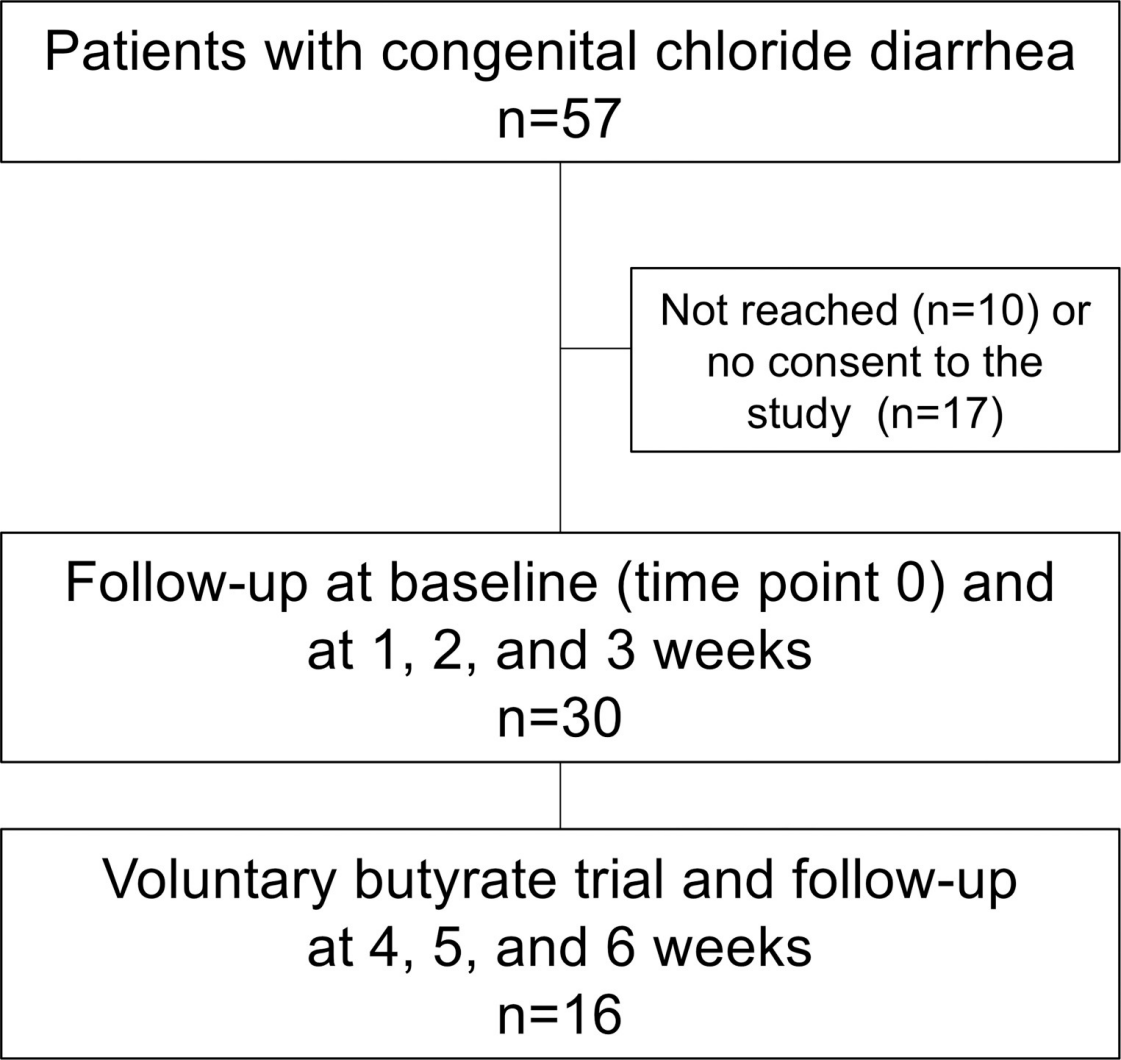
Study participant selection and follow-up.

The flowchart of study participant selection is shown in **Fig 1**.

Inclusion criteria were a diagnosis of CLD, age 2–60 years, and possibility to collect fecal samples once a week. Exclusion criteria were infection at the study entry or use of antibiotics during the previous 3 months. A total of 57 potential participants were selected based on a previous study of the Finnish CLD cohort [10] and from the records of the Finnish university hospitals. Altogether 47 patients were reached and received written information about the study by the study personnel of the Children´s Hospital (University of Helsinki). A total of 30 patients were interested in participating and gave their (or parents for the minors) written informed consent. Of these 30 study participants, 16 further agreed to participate in a voluntary trial with oral butyrate. The study was conducted in 2018 and was supervised by the Children´s Hospital. Study protocol was approved by the ethics committee of the Hospital District of Helsinki and Uusimaa (HUS/895/2017). Study was registered 17 Jan 2018 in the online Research Register of Helsinki University Hospital (registration number HUS/185/2018). At the time of registration, no further registration to international registries was requested. Research was performed in accordance with the Declaration of Helsinki and in accordance with the relevant guidelines and regulations.

Two previously collected cohorts were utilized as controls in fecal microbiota analyses: 43 subjects (median age 20.2 years, interquartile range (IQR) 14.4–31 years; 27 males) with Crohn’s disease (CD) in clinical remission (median fecal calprotectin 203 ug/g), and 19 healthy controls (median age 21.8 years, IQR 20.6–27.3 years; 10 males) [32]. All patients with CD presented with pediatric onset disease, and 80% had ileocolonic disease.

### Study protocol and samples

All 30 participants were examined at the study entry (time point 0, baseline). Data on participants’ health, intestinal symptoms, and diet were recorded, and quality of life assessed [33], as detailed in the study protocol (**S1 File**) and supplementary methods (**S1 Methods**). Their blood, urine and fecal samples were collected. Thereafter, the patients provided fecal samples once a week after 1, 2, and 3 weeks (time points 1, 2, and 3). After the 3-week follow-up of 30 patients, 16 of them participated in a voluntary trial with oral butyrate. One capsule (BioCare® Butyric Acid; Biocare Ltd, Birmingham, UK) contained 605 mg of butyric acid. Targeted dose of butyrate was 100 mg/kg/day, divided in two daily doses, for 3 weeks. The subjects attending the butyrate trial were followed up for another 3 weeks and they collected fecal samples once a week at 4, 5, and 6 weeks from the baseline visit (**Fig 1**). Data on participants’ health and intestinal symptoms were recorded during and after the butyrate trial as detailed in the supplementary methods (**S1 Methods**). After the butyrate trial, blood and urine samples were collected.

Fecal samples from the control cohorts (CD and healthy controls), and data on their dietary intake of nutrients, had been collected previously [34]. These control samples were subjected to fecal microbiota analyses in parallel with fecal samples obtained from the CLD patients.

### Laboratory analyses

Blood tests included plasma levels of sodium, chloride, and potassium, venous blood gas analysis, blood count, creatinine, cystatin C, urea, and C-reactive protein (CRP). Urine level of chloride, glomerular filtration rate (GFR), and fecal calprotectin were measured. All these measurements were performed by the laboratories of the university hospitals.

After home-based fecal sample collection, the samples were immediately put in the freezer (-20°C) and thereafter, were transferred into our research laboratory and stored long-term at -70°C. Fecal samples were prepared for microbiota analyses as detailed below. Moreover, we performed analyses for water content, total protein, intestinal alkaline phosphatase (IAP) activity, total immunoglobulins, and methylglyoxal-derived hydroimidazolone-1 (MG-H1) (S1 Methods).

### DNA extraction and microbiota analysis

Processing of fecal samples (CLD, CD, healthy controls) was performed by the same personnel and according to the standard protocols of the same laboratory. DNA extraction was performed from by the repeated bead beating method using the KingFisherTM Flex automated purification system (ThermoFisher Scientific, USA). Due to liquid nature of the CLD samples, these samples were vortexed to dispense the solids and pipetted directly to the lysis buffer instead of suspending the samples first to 1 mL of sterile PBS. DNA was quantified using Quanti-iT™ Pico Green dsDNA Assay (Invitrogen, San Diego, CA, USA). Generation of the 16S rRNA gene amplicons (primers 341F 5′-CCTACGGGNGGCWGCAG-3′ and 785R 5’-GACTACHVGGGTATCTAATCC-3′) for Illumina MiSeq sequencing was performed as described previously [35]. The V3-V4 amplicons, equipped with Illumina TruSeq dual index primers (PE-121-1003), were sequenced with Illumina MiSeq (2 × 300 bp reads) using the MiSeq v3 reagent kit (MS-102-3003) with 5% PhiX as spike-in (Illumina). Sequencing data were processed and analyzed using the R package mare [36], utilizing USEARCH for read processing, operational taxonomic unit (OTU) clustering, and taxonomic data annotation [37]. The annotation was based on the reads, while OTUs (clustered at 97% similarity) were used only for the calculation of microbial richness. The success of all runs and analyses was controlled by internal reference samples and mock controls [38], and the Silva database was used for taxonomic annotation [39]. In data analyses, the number of reads was used as an offset in all statistical models, without rarefaction or transformations [35].

### Statistical analysis

Statistical analyses were performed using the R package mare [36]. Diversity was calculated as the inverse Simpson diversity index and richness as the number of OTUs. Samples with <2000 reads were excluded from the analyses. Unsupervised Principal-coordinate Analyses (PcoA) were conducted using the Bray-Curtis dissimilarity as the distance measure and calculated with the capscale function and the Bray-Curtis dissimilarities with the function vegdist of the R package vegan [40]. GroupTest function of the mare package was used for comparison of the relative abundances of bacterial genera between the groups. The function selects the most optimal model for each taxon based on its distribution, using either the glm.nb function from the MASS package [41], lm function from base R with log-transformation if necessary, or the gls function from the nlme package [42]. The GroupTest function calculates a model that is appropriate for each taxon separately and attempts to find a suitable model for the taxon. When no suitable model is found, and the model assumptions are not met, no *P* value is reported for the taxa. The total read counts per sample were used as the offset in the models. In statistical analyses, the healthy control group was used as the reference group, as stated in the results section. We tested microbiota differences between CLD subjects and healthy controls, CLD and CD patients, and between CD and healthy controls as described. Standard Benjamini-Hochberg corrections for false discovery rate (FDR) were applied. FDR-corrected *P* values of <0.1 were considered significant, and unadjusted *P* values are reported in the text. In the Figs demonstrating differences between the groups (CLD, CD, healthy) for individual taxa, we show only those with an unadjusted *P* value of <0.01. Details of statistical tests can be found in the figure legends and in the results section. Fecal biomarkers were compared by the Mann-Whitney *U* test and Wilcoxon matched-paired signed rank test as detailed in the footnotes of the tables.

## Results

### Baseline clinical characteristics of patients with CLD

This study involved 30 subjects with CLD aged 2–53 (median, 31) years. All patients were homozygous for the Finnish founder mutation of the *SLC26A3* gene [2]. They had regular salt substitution therapy with a median dose of chloride 3 mmol/kg/day, being compatible with the recommendation of 3–4 mmol/kg/day [9]. None of the participants reported previous or current use of butyrate.

These 30 patients underwent clinical examination and laboratory testing at the study entry (**S1 Table**). None reported recent or current use of antibiotics. All had watery stools. Daytime stool frequencies were: 0–2 (in 3% of patients), 3–5 (43%), 6–8 (40%), and >9 (13%) stools/day. The median score for abdominal symptoms was low of 2 on the visual analog scale (VAS) of 1 to 7. Altogether, 15 (50%) of the patients reported no abdominal pain for CLD. The patients reported their overall quality of life as well as physical, emotional and social functioning as good (VAS medians of all these items 6 on the scale of 1 to 7). Urine chloride excretion was over the detection limit of 20 mmol/L in 20 (67%) out of 30 patients, indicating adequate substitution of fecal electrolyte losses. None of the patients had signs of chronic volume depletion, metabolic alkalosis, or kidney disease. However, hypokalemia (range, 2.1–3.2 mmol/L) was observed in 6 (20%) of the subjects, as a potential indicator of inadequate salt substitution.

All patients had uneventful clinical course during the 3-week follow-up with the standard salt substitution, during which they provided fecal samples once a week. For most of the subjects, fecal calprotectin levels were normal (<100 μg/g) and there were no signs of systemic inflammation or anemia. However, this series involved 3 adults (aged 30–40) with IBD. One with CD was in remission and had low levels of calprotectin (<5 μg/g). Two patients had throughout the study elevated levels of fecal calprotectin (mean levels of 658 and 930 μg/g), anemia (hemoglobin levels of 83 and 130 g/l), and elevated CRP levels (31 mg/L in both). They were considered to have undiagnosed IBD and got their diagnoses after this study. These 3 subjects with CLD-associated IBD were assessed as one separate subgroup corresponding CLD-IBD in fecal microbiota analyses. One adult patient was diagnosed with sacroiliitis and had slightly elevated calprotectin values (mean 315 μg/g) without clinical suspicion or diagnosis of IBD. An adolescent had a diagnosis of type 1 diabetes. Other chronic diseases were not detected in our series.

### Fecal microbiota in CLD is distinct from that of healthy subjects and CD

A total of 165 fecal samples were collected, and 129 fecal samples included in final fecal microbiota analyses, after excluding 16 children’s samples and 20 samples for the low read count (<2000) or processing/technical error. The microbiota in CLD children (n = 3; <7 years old) were different from adult CLD samples (**S1 Fig**) in agreement with age-related differences in gut microbiota composition [43]. Therefore, children’s samples were excluded from the final analyses. In contrast, adolescent samples from CLD subjects were highly similar to adult samples in terms of microbiota composition (**S2 Fig**) and were included in the analyses.

**Fig 2:**
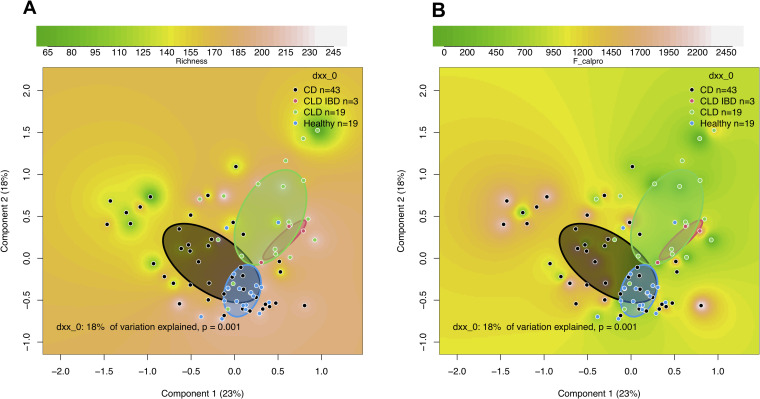
Comparison of fecal microbiota in congenital chloride diarrhea (CLD), CLD-associated inflammatory bowel disease (CLD IBD), Crohn’s disease (CD), and healthy controls. Shown are Principal-coordinate Analysis (PcoA) plots based on Bray-Curtis dissimilarities of the samples, showing as background (A) richness of the microbiota and (B) fecal calprotectin levels for the samples. The units of richness in PcoA plots are arbitrary. Clusters are shown by circles, which were drawn based on the standard deviations of the data points in each category of the samples (CD, CLD IBD, CLD, Healthy).

Fecal microbiota in CLD subjects at the study entry, analyzed by 16S rRNA gene amplicon sequencing, were compared to the microbiota of healthy subjects (n = 19) and those with CD (n = 43). The median read counts were comparable in these groups (43,325 reads in CLD, 53,838 in healthy subjects, and 51,975 in CD). While the mean microbial richness was equal in CLD and healthy subjects, microbial diversity, as measured by the inverse Simpson diversity index, was higher in CLD compared with the healthy controls (*P* = .004) and those with CD (P < .001; **S2 Table**). The composition of microbiota was highly similar between the CLD subjects but clustered clearly distinct from the microbiota of healthy subjects and subjects with CD as visualized by PCoA (**Fig 2A**). In those 3 CLD subjects with IBD, we found sub-clustering of their gut microbiota, still resembling that of CLD (**Fig 2A**). The differences between CLD patients and healthy controls were not related to fecal calprotectin levels (**Fig 2B**). Even though only the subjects with the lowest calprotectin levels of <50 ug/g were considered (**S3 Fig**), the groups (CLD, CD, healthy) still differed from each other.

**Fig 3:**
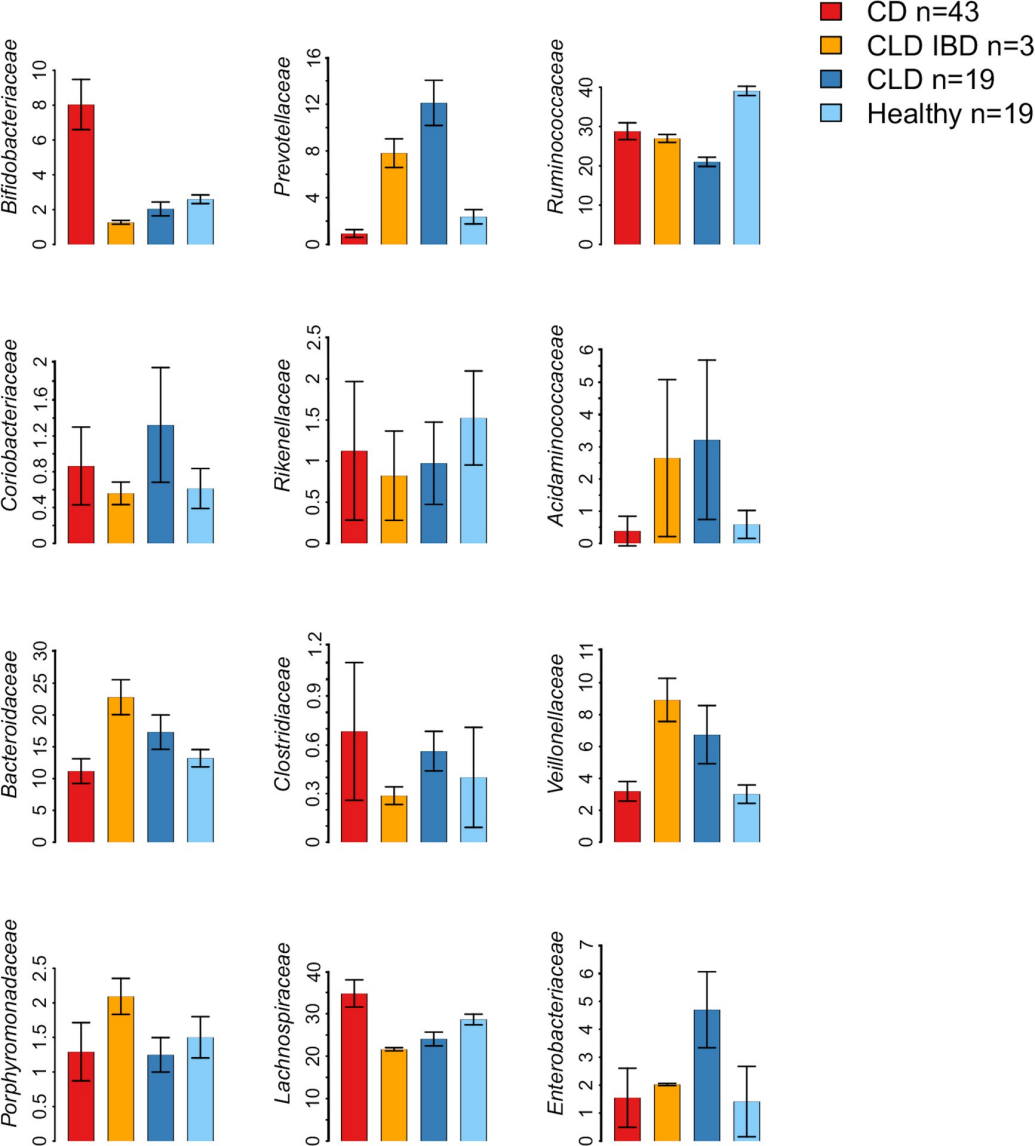
Microbiota composition in congenital chloride diarrhea (CLD), CLD-associated IBD (CLD IBD), Crohn’s disease (CD), and healthy controls. Shown are family-level microbiota composition for the taxa with significant differences (*P*<0.01) between CLD and healthy subjects. Data are presented as group means and standard errors of the mean. Detailed data are presented in **S3 Table**.

While normal adult gut microbiota is dominated by *Ruminococcaceae* and *Lachnospiraceae* [44], we observed lower abundances of these dominant taxa in both CLD and CLD-associated IBD (**Fig 3**). The most significant differences in the microbiota composition between CLD and healthy individuals were characterized by the increased relative abundance of Proteobacteria, both in CLD (3.4 fold increase; *P* < .001) and in CLD-associated IBD (1.5 fold increase; *P* < .001) (**Fig 3**; **S3 Table**). As the most abundant genus in the Proteobacteria group, we observed increased relative abundance of *Escherichia* in CLD (45.7 fold increase; *P* < .001) and in CLD-IBD (12.7 fold increase; *P* < .001). Of other bacterial genera, *Veillonella* was abundant in CLD (9.9 fold increase; *P* < .001) and in CLD-associated IBD samples (11.6 fold increase; *P* < .001). Similarly, *Prevotella* showed increased relative abundances in CLD (5.1 fold increase; *P* < .001) and in CLD-associated IBD (3.5 fold increase; *P* < .001). *Lachnospiraceae* were less abundant particularly in those 3 subjects with CLD-associated IBD (0.76 fold change; *P* < .001), and less pronounced in other CLD subjects (0.84 fold change; *P* < .001), in comparison with healthy individuals (**Fig 3**; **S3 Table**). When CLD samples (without IBD) were compared with CD samples, the most significant differences were the increased relative abundances of *Prevotella* (13.0 fold increase; *P* < .001), *Veillonella* (1.9 fold increase; *P* = 0.01), Proteobacteria (3.1 fold increase; *P* < .001) and *Acidaminococcus* (7.7 fold increase; *P* = 0.002) in CLD, and lower relative abundances of *Lachnospiraceae* (0.71 fold change; *P* < .001) in CLD (**Fig 3**).

### Diet modulates fecal microbiota in CLD

Next, we studied microbiota differences in relation to the dietary intake of nutrients between CLD subjects and healthy individuals. Overall, we found that the composition of diet in subjects with CLD was non-optimal as they consumed less fiber, but more sucrose and saturated fats than recommended (**S4 Table**). In addition, the median intake of vitamin C, vitamin D and folate were suboptimal (**S5 Table**), descriptive of an overall poor diet quality.

**Fig 4:**
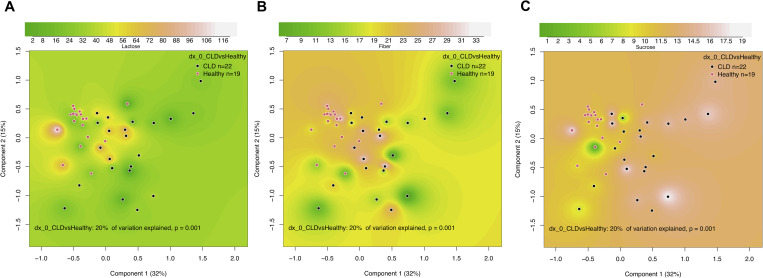
Effects of diet on fecal microbiota in congenital chloride diarrhea (CLD) and healthy controls. Shown are Principal-coordinate Analysis (PcoA) plots based on Bray-Curtis dissimilarities of the microbiota in relation to dietary (A) lactose (grams/day), (B) fiber (grams/day), and (C) sucrose (% of energy intake). The units of richness in PcoA plots are percentages.

The fecal microbiota of CLD was associated with the dietary intake of lactose, fiber, and sucrose (**Fig 4**). Notably, we observed that those CLD subjects with higher fiber and lactose intake had fecal microbiota resembling that of healthy subjects. In contrast, high sucrose intake was associated with a highly abnormal fecal microbiota in CLD. However, the changes in the gut microbiota in association with low lactose (**S6 Table**) or fiber intake (**S7 Table**), and high sucrose intake (**S8 Table**), were diverse and we could not identify specific genera associated with these dietary patterns. Dietary protein, fat, or total carbohydrates showed no significant association with the overall fecal microbiota composition or individual taxa in CLD.

### Butyrate has a minor modulatory effect on microbiota in CLD

Altogether 16 subjects participated in the observational butyrate trial and follow-up for additional 3 weeks. The subjects took 3 to 6 (median, 4) capsules daily, with the total dose of butyrate ranging from 1815 to 3630 mg/day (median 2420 mg/day) for 3 weeks. The median dose of butyrate was 36 mg/kg/day (range from 31 to 42 mg/kg/day). These doses of butyrate were under the targeted dose of 100 mg/kg/day but close to the daily dose of 3 capsules for adults (butyrate 1815 mg/day; 30 mg/kg/day for a subject weighing 60 kg), as recommended by the manufacturer.

Only two out of 16 patients, both non-IBD subjects, found that butyrate reduced their diarrhea and had beneficial effects on their intestinal symptoms. One of the two subjects with CLD-associated IBD, who took part in the butyrate trial, experienced subjective benefit from butyrate through alleviation of her symptoms of joint pain. However, 4 patients had poor compliance and reported variable doses of butyrate (n = 1) or stopping butyrate after 11–14 days (n = 3) because of worsening of diarrhea or abdominal pain.

**Fig 5:**
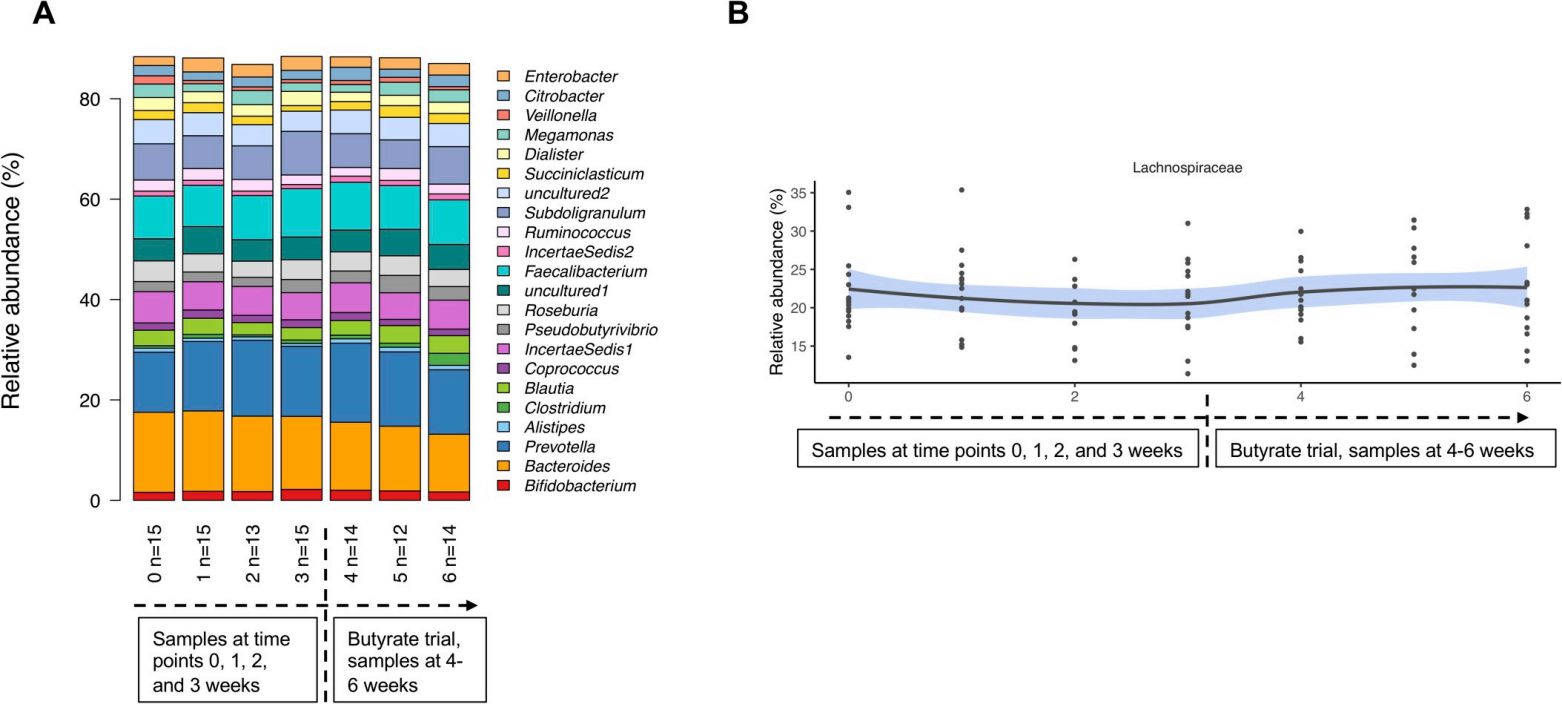
Effects of butyrate on microbiome in subjects with congenital chloride diarrhea (CLD). Shown are (A) clustered stacked column graphs demonstrating microbiota differences at the genus level at the study entry (time point 0) and further during the standard salt substitution (time points 1, 2, and 3 weeks), and during the butyrate trial (time points 4, 5, and 6 weeks), and (B) microbiota changes for *Lachnospiraceae* at the family level at the different time points (*P*<0.01; CovariateTest for the changes after starting the administration of butyrate). The line defines mean and shaded area 95% confidence intervals.

The overall microbial richness and diversity showed no differences during or after butyrate treatment, when compared with the samples taken during the standard salt substitution therapy (**S4 Fig**). Butyrate showed only mild modulatory effects on fecal microbiota (**Fig 5A**). However, butyrate changed the microbiota toward slightly increased relative abundance of *Lachnospiraceae* (**Fig 5B**), particularly after 1 and 2 weeks from starting the butyrate (**S9 Table**). At the end of the 3-week trial, we observed increased relative abundance of Bacteroidales group *Rikenellaceae* (difference in delta between treated and not treated; *P* < .001) and decreased relative abundance of *Ruminococcaceae* (P = 0.002) (**S9 Table**; **S5 Fig**). During the 3-week butyrate trial, major changes in fecal microbiota composition within the butyrate group were seen in Firmicutes order as an increased relative abundance for *Lactobacillales* (P = 0.003) and genus *Blautia*, a member of the *Lachnospiraceae* (*P* < .001) (**S10 Table**). In contrast, during the standard salt substitution therapy, no major changes in fecal microbiota between the first and the last sample from the same individual occurred, except for the slight variation for *Ruminococcaceae* (**S9 and S10 Tables**). Intestinal symptoms showed no major changes before or during the butyrate trial (**S6 Fig**). Moreover, laboratory tests for electrolyte and acid-base balance showed no changes after the butyrate trial (**S11 Table**).

### Analyses of fecal biomarkers

We searched for markers potentially reflecting intestinal inflammation and gut microbial homeostasis or the effects of butyrate in CLD. Stool samples were subjected to analyses of water content, total protein, IAP activity, total immunoglobulins, and MG-H1. Compared with in-house reference data from healthy controls [45], CLD stool samples had high water (>90%) and low protein content, and increased IAP and IgA concentrations (**Table 1**). None of these biomarkers distinguished CLD and CLD-associated IBD. Neither these fecal biomarkers nor calprotectin, which was elevated in association with IBD, showed significant changes during the follow-up or oral administration of butyrate (**S7 Fig**; **S12 Table**).

**Table 1:** Levels of fecal biomarkers in congenital chloride diarrhea (CLD) and healthy controls.

	CLD	Healthy controls	*P* value
Subjects (M/F)	30 (12/18)	41 (20/21)	
Age (years)	28 ± 13	37 ± 11	0.03
Fecal protein (mg/ml)	1.1 ± 0.2	1.7 ± 0.6	<0.001
IAP (U/l)^1^	459 [290–726]	195 [114–1531]	
IAP (U/l) /protein^1^	406 [288–608]	136 [80–885]	0.01
IgA (μg/ml)^1^	16.5 [2.3–47.3]	6.8 [2.0–10.6]	0.01
IgA (μg/protein)^1^	12.3 [2.1–43.7]	3.7 [1.4–7.0]	0.001

Fecal sample collections: CLD (data from four samples from each individual, collected at time points 0, 1, 2, and 3 weeks during the standard salt substitution). Healthy controls (data from two samples from each individual collected during 1 week). The data are presented as mean ± SD or median [interquartile range]. IAP, intestine alkaline phosphatase. Only *P*<0.05 are shown (Mann-Whitney *U* test).

## Discussion

This study demonstrates that the loss of functional SLC26A3 in CLD is associated with alterations in the relative abundance of major taxa of the fecal microbiota. We found enrichment of *Escherichia*, *Veillonella*, and *Prevotella*, and depletion of the normally dominant taxa *Ruminococcaceae* and *Lachnospiraceae* in CLD compared with healthy controls. Unexpectedly, the microbiota in all patients with CLD were highly similar, irrespective of the presence of IBD (including pre-diagnostic samples). As for butyrate, low-dose oral supplementation was poorly tolerated and showed no major effects on diarrhea or microbiota. However, a major butyrate-producing bacterial taxon *Lachnospiraceae* was less abundant in CLD. Furthermore, CLD-associated changes in microbiota were partly attributable to a diet low in fiber and high in sugar but did not explain the association to IBD.

In rare diseases such as CLD, clinical studies have many challenges and potential confounders. Finland has one of the world’s highest incidences of CLD (1: 30 000 to 1: 40 000), 1 or 2 new patients born annually, and currently around 60 living patients using the salt substitution introduced in our country in the late 1960s [8, 9]. Thus, a major strength of this study is the sample size of 30 patients with the same genetic background [1], regular salt substitution, similar diarrheal symptoms, and normal electrolyte and acid-base balance without signs of renal insufficiency. Moreover, our set included 3 patients with IBD, which allowed assessment of fecal microbiota in this major comorbidity of CLD. As additional strengths, we included altogether 129 fecal samples from 23 CLD subjects in microbiota analyses and performed parallel analyses of fecal samples to compare the gut microbiota in CLD with both that of healthy subjects and patients with CD. We excluded children from microbiota analyses for the strong age-related differences in gut microbiota composition [43] and because samples from healthy control children were not available. Despite the overall modest sample size, the distinct clustering of microbiota in CLD was strikingly evident. This might reflect both the role of gut function, such as transit time and watery stool content modulating the gut microbiota [46], and the associated dietary differences between CLD patients and healthy controls. Even though no changes in microbiota characterized CLD-associated IBD, the number of IBD cases was too low to draw general conclusions. Given that data on genotype-phenotype differences in CLD are lacking [2], our findings might be generalizable to all CLD subjects who receive adequate salt substitution. However, the targeted doses of butyrate were not reached, which might underestimate potential effects of butyrate on the microbiota.

The observed aberrant composition of gut microbiota in CLD could be explained by several mechanisms. First, the rapid transit time likely directly increases the relative abundance of bacteria from the small intestine in feces, which may be associated with increased oxygen levels in the colon and reduce the abundance of strictly anaerobic taxa, such as *Lachnospiraceae* and *Ruminococcaceae* [47, 48]. Second, impaired sodium chloride and fluid reabsorption may induce alterations in the microbiota [24, 25], and promote elevated levels of Bacteroidetes [49] that we observed for *Prevotella* in association with CLD. Third, SLC26A3 deficiency may cause defects in the firm mucus layer of the colon and thereby modulate microbiota composition [26, 27]. However, we found no increase in microbiota richness in CLD, previously linked to lose stools [46], or suppression of *Lactobacillus* species, the alteration associated with a high salt diet [24, 25]. In contrast, the CLD-related patterns of the low abundance of Firmicutes taxa *Lachnospiraceae* and *Ruminococcaceae*, and an increase in Proteobacteria, are consistent findings in IBD [50, 51]. Similarly, higher abundances of Proteobacteria and the Gram-negative Firmicutes-family taxon *Veillonella*, which we observed in CLD, have been associated with colitis in mice [52] and with human CD [53], respectively. Moreover, the significant increase in *Escherichia*, as observed here for CLD, is a finding previously linked to CD development [54]. ln contrast, the relative abundance of Faecalibacterium, being potentially protective against IBD [55], showed no differences between CLD and other groups (**S8 Fig**). Although our results support that the microbiota in CLD might be associated with proinflammatory changes, we found neither evidence of increased inflammation in CLD patients nor microbiota alterations specific to CLD-associated IBD. Furthermore, the fecal microbiota in CLD did not strongly resemble that of CD patients but showed a distinct CLD-specific pattern. Collectively, the observed microbiota alterations in CLD likely contribute to mucosal degradation, weakened gut barrier, and increased proinflammatory immune stimulation, due to the reduced relative abundance of anti-inflammatory and butyrate-producing taxa, such as *Lachnospiraceae* and *Ruminococcaceae* [56], and increased relative abundance of lipopolysaccharide-producing taxa such as *Escherichia* [57].

The association between defective SLC26A3 function and IBD is markedly strong. In CLD, the prevalence of IBD in adolescent or adult subjects is as high as 18% in European cohorts [13]. In rodents, slc26a3-deficiency has been repeatedly linked to IBD [12, 26, 27]. Notably, the high microbial diversity in SLC26A3-deficient humans as presented here is not consistent with the observations of low diversity gut microbiota of both slc26a3-deficient [26] and nhe3-deficient mice [58]. Whether aberrant microbial composition in human CLD is a cause or consequence of the intestinal mucosa impairment remains a major question for future studies, also wider in the context of IBD [59]. Future studies should identify long-term individual differences in mucosal health between CLD patients who develop or do not develop IBD, to design potential preventive actions.

The composition of diet was non-optimal in our patients with CLD and significantly associated with the microbiota composition. High dietary sucrose intake showed association with abnormal microbiota. In contrast, a diet rich in fiber associated with healthier microbiota in CLD. The beneficial effects of fiber are likely to be mediated by the increased production of SCFAs, specifically butyrate, through microbial fermentation in the intestine. Although butyrate is unlikely to directly affect the microbiota, it might improve gut epithelial function and reduce inflammation, which in turn modulates the microbiota [28, 60]. In agreement with this, gut microbiota remained mostly unchanged during the administration of oral butyrate. However, the slight increase in the abundance of *Lactobacillales* and *Lachnospiraceae Blautia* might reflect small but potentially beneficial effects of butyrate in the colon. While unanswered questions of the efficacy of butyrate in CLD remain, only 2 out of 16 subjects in our study reported that butyrate alleviated their symptoms of CLD. The overall poor compliance with butyrate supplementation in this study, together with the weak previous evidence [29–31], discourages further trials with this therapy. Whether dietary changes including a diet rich in fiber, thereby potentially enhancing normal gut microbiota, modulate intestinal health in CLD is an intriguing question.

We tested the possibility that fecal biomarkers other than calprotectin would indicate intestinal inflammation or the effects of butyrate but found no such evidence. IAP [45, 61, 62] and total IgA, which plays an important role in the mucosal immune defence [63], were higher in CLD than in the reference population. However, these biomarkers were not related to the CLD-associated IBD in those few subjects. Therefore, our study fails to provide new screening tools, which would help to diagnose or predict IBD in subjects with CLD.

In conclusion, this study shows evidence of gut microbiota dysregulation in patients with CLD and thus highlight the link between intestinal anion exchange defect of SLC26A3 and the fecal microbiota. However, although the microbiota in CLD was different from that of healthy subjects or patients with Crohn´s disease, we observed no major differences in CLD and CLD-associated IBD. Short chain fatty acid supplementation with butyrate failed to induce detectable modulation of the microbiota. Importantly, further studies on the mechanisms that modulate the microbiota, mucosal barrier function and intestinal inflammation in rare disorders such as CLD might reveal common mechanisms and risk factors for the development of human IBD.

## Supporting information

S1 File Study protocol. (DOCX)

S1 Methods Supplementary methods. (PDF)

S1 Fig Shown are clustered stacked column graphs demonstrating microbiota differences at the genus level for adults (including 3 adolescent subjects) and children (n = 3) with congenital chloride diarrhea (CLD). (TIFF)

S2 Fig Comparison of gut microbiota in adults with congenital chloride diarrhea (CLD; n = 19) and in 3 adolescent subjects (1, 2, and 3) with CLD aged 12–18 years. Shown are Principal-coordinated Analysis (PcoA) based on BrayCurtis dissimilarities of the samples (A) in all samples of the subjects, and (B) only in the samples taken at the study entry. The number of CLD samples in the final microbiota analyses and taken during the standard treatment were: 22 (time point 0, baseline), 23 (time point 1 week), 21 (time point 2 weeks), and 23 (time point 3 weeks). During the butyrate trial, fecal microbiota were further analyzed from 14 (time point 4 weeks), 12 (time point 5 weeks), and 14 (time point 6 weeks) samples.
(TIFF)

S3 Fig Comparison of gut microbiota between the subjects with the lowest levels of calprotectin (<50 ug/g) in different groups. Shown are (A) Principal-coordinated Analysis (PcoA) based on Bray-Curtis dissimilarities of the richness of the microbiota, and (B) clustered stacked column graphs demonstrating microbiota differences at the genus level. The clusters in (A) are shown by circles, which were drawn based on the standard deviations of the data points in each category of the samples (CD, Crohn’s disease; CLD, Congenital chloride diarrhea; Healthy).
(TIFF)

S4 Fig Comparison of gut microbiota between the subjects with CLD at each data points. Shown are Principal-coordinated Analysis (PcoA) based on Bray-Curtis dissimilarities of the richness of the microbiota at the study entry (time point 0) and further during the standard salt substitution (time points 1, 2, and 3 weeks), and during the butyrate trial (time points 4, 5, and 6 weeks).
(TIFF)

S5 Fig Covariate plots showing fecal microbiota composition before the butyrate trial (time point 3 weeks), and as measured once a week during the butyrate trial (time points 4, 5, and 6 weeks) in the subjects with congenital chloride diarrhea (CLD). Shown are microbiota composition for the taxa with significant differences (P<0.01) (CovariateTest) between the first and last measurement (time point 3 vs 6). Similar data for *Lachnospiraceae* is shown in Fig 5B of the main article. Lines define group means and shaded areas 95% confidence intervals.
(TIFF)

S6 Fig Gastrointestinal (GI) symptoms of the study subjects (n = 30) during the study. Treatment group indicates the group of subjects who attended the 3-week butyrate trial. No treatment defines the group with the standard salt substitution therapy and follow-up period of 3 weeks. Lines define the mean values and whiskers standard deviations.
(TIFF)

S7 Fig Fecal biomarker levels in individuals with congenital chloride diarrhea (CLD) treated with butyrate. Line graphs show biomarker levels from the baseline to 6 weeks at the end of the follow-up period. Data are presented as mean with standard errors of mean. IAP, intestinal alkaline phosphatase. MG-H1, methylglyoxal-hydro-imidazolone. Shaded areas define samples taken during the butyrate treatment.
(TIFF)

S8 Fig Shown are microbiota composition for Faecalibacterium in each category of the samples (CD, Crohn’s disease; CLD IBD, Congenital chloride diarrhea and inflammatory bowel disease; CLD, Congenital chloride diarrhea; Healthy). Data are presented as group means and standard errors of the mean.
(TIFF)

S1 Table Characteristics of the subjects and laboratory values at the study entry. (PDF)

S2 Table Differences in richness and diversity of fecal microbiota between the groups. (PDF)

S3 Table Microbiota differences. *P* values for microbiota differences in congenital chloride diarrhea (CLD), CLD-associated IBD (CLD IBD), and Crohn’s disease (CD), in comparison with the microbiota of healthy controls (n = 19). Shown are only the taxa with adjusted *P* values (FDR_p) <0.1 after comparison of the subgroup (CD, CLD IBD, or CLD) and healthy controls (GroupTest).
(PDF)

S4 Table Nutrient intake. Daily intakes of energy and energy-yielding nutrients in patients with congenital chloride diarrhea (CLD; n = 30). Values outside the reference range are shown bolded.
(PDF)

S5 Table Vitamins, minerals and trace elements. Daily intakes of vitamins, minerals and trace elements in patients with congenital chloride diarrhea (CLD; n = 30). Values outside the reference range are shown bolded.
(PDF)

S6 Table Microbiota and lactose intake. *P* values for fecal microbiota composition in relation to dietary lactose (grams/day) in congenital chloride diarrhea (CLD; n = 22) and healthy controls (n = 19). Shown are only the taxa with adjusted *P* values (FDR) <0.1 (CovariateTest). p, *P* value. FDR, adjusted *P* value after Benjamini-Hochberg correction.
(PDF)

S7 Table Microbiota and fiber intake. *P* values for fecal microbiota composition in relation to dietary total fiber (grams/day) in congenital chloride diarrhea (CLD; n = 22) and healthy controls (n = 19). Shown are only the taxa with adjusted *P* values (FDR) <0.1 (CovariateTest). p, *P* value. FDR, adjusted *P* value after Benjamini-Hochberg corrections.
(PDF)

S8 Table Microbiota and sucrose intake. *P* values for fecal microbiota composition in relation to dietary sucrose (% of energy intake) in congenital chloride diarrhea (CLD; n = 22) and healthy controls (n = 19). Shown are only the taxa with adjusted *P* values (FDR) <0.1 (CovariateTest). p, *P* value. FDR, adjusted *P* value after Benjamini-Hochberg correction.
(PDF)

S9 Table Microbiota changes during butyrate. *P* values for the changes in fecal microbiota composition during the 3-week follow-up. Data are presented separately for the group with the standard salt substitution treatment (treatment_standard) and the butyrate trial group (treatment_butyrate). For those with the standard salt substitution, *P* values are shown for the differences between 1, 2, and 3 weeks (p_1, p_2, and p_3) vs baseline (time point 0). For the butyrate trial group, *P* values are shown for the differences during the butyrate trial at 1, 2, and 3 weeks (p_1, p_2, and p_3) versus the beginning of the trial. Shown are only the taxa with adjusted *P* values (FDR) <0.1 after ChangeTest. p, *P* value. FDR, adjusted *P* value after Benjamini-Hochberg correction.
(PDF)

S10 Table Microbiota changes after butyrate. *P* values for the changes in fecal microbiota between the baseline and the end of the 3-week follow-up (time3). Data are shown for the group with the standard salt substitution (treatment_standard; 0 weeks vs 3 weeks), and for the group attending the butyrate trial before the trial (before_butyrate; 0 weeks vs 3 weeks), and during the trial (after_butyrate; before butyrate vs after 3 weeks administration of butyrate). Shown are only the taxa with adjusted *P* values (FDR) <0.1 after CovariateTest. p, *P* value. FDR, adjusted *P* value after Benjamini-Hochberg correction.
(PDF)

S11 Table Characteristics of the study groups. No significant differences were detected between the groups. Samples from 15 out of 16 subjects attending the butyrate trial were available before and after the trial.
(PDF)

S12 Table Biomarkers. Levels of fecal biomarkers in patients with congenital chloride diarrhea (CLD) before and after butyrate treatment. Samples from 15 out of 16 subjects attending the butyrate trial were available before and after the trial.
(PDF)
