# Supplementary material for: Fecal microbiota in congenital chloride diarrhea and inflammatory bowel disease
Source: PLoS One. 2022 Jun 9;17(6):e0269561. doi: 10.1371/journal.pone.0269561 (PMC9182261; doi:10.1371/journal.pone.0269561)
Supplement: S1 File — (DOCX) [file pone.0269561.s001.docx]

**Study Protocol**

(22 Jan 2018, Version 10)

Registered in the online Research Register of Helsinki University Hospital 17^th^ Jan 2018 (registration number HUS/185/2018)

**Study Title:**

Gut Microbiota in Patients With Congenital Chloride Diarrhea

**Principal Investigator:**

Kaija-Leena Kolho, MD, PhD

Children´s Hospital

University of Helsinki

Stenbäckinkatu 11

FI-00029 Finland

email: [kaija-leena.kolho@helsinki.fi](mailto:kaija-leena.kolho@helsinki.fi)

gsm: +358 50 566 1442

**Supported by:**

Pediatric Research Foundation

Helsinki University Hospital Grants (no. TYH2018212 and TYH2020217)

**I Study Objectives**

**Primary Objective**

To describe the gut microbiota in patients with congenital chloride diarrhea (CLD).

**Secondary Objectives**

To study the effects of oral butyrate on the diarrhea and the gut microbiota.

**II Background and Rationale**

**Background on Disease**

A rare autosomal recessive disease congenital chloride diarrhea (CLD; OMIM #214700) is caused by mutations in the *solute carrier family 26 member 3* (*SLC26A3* alias *DRA*) gene on chromosome 7q22.3-31.1 ^1,2^. *SLC26A3* encodes for a major apical epithelial chloride-bicarbonate exchanger of the terminal ileum and colon ^3^. While options to resolve chronic diarrhea are missing, oral salt substitution with sodium and potassium chloride allows favorable outcome in CLD and prevents complications such as chronic volume and electrolyte depletion and kidney disease ^4,5^.

**Study Rationale**

For unknown reason, CLD subjects are prone to inflammatory bowel disease (IBD) ^4^. Whether gut microbiota is affected in CLD, or plays a role in CLD-associated IBD, remains unknown. We and others previously found that oral administration of short-chain fatty acid (SCFA) butyrate, the most essential end-product of bacterial carbohydrate fermentation in the colon and the principal nutrient for colonocytes ^6^, reduces the diarrhea in some patients with CLD ^7-9^. While these individual responses are poorly understood, luminal butyrate might increase intestinal sodium chloride and fluid reabsorption by modulating colonic epithelial ion transport ^6^. Collectively, these data prompted us to design this study on the gut microbiota and the potential modulatory effects of butyrate in patients with CLD.

**III Study Design**

This prospective cohort study involves the following parts:

1. Study visit, laboratory testing, and recording of intestinal symptoms, quality of life, and food diary
2. 3-week follow-up for all participants and collection of fecal samples once a week
3. 3-week voluntary trial with oral butyrate and collection of fecal samples once a week

**IV Selection and Enrolment of Participants**

**Background**

Potential participants will be selected among the Finnish CLD patients based on previous studies of the CLD cohort and from the records of the university hospitals.

**Inclusion Criteria**

Inclusion criteria are: age 2-60 years and the possibility to collect fecal samples once a week.

**Exclusion Criteria**

Exclusion criteria are: infection at the study entry or use of antibiotics during the previous 3 months.

**V Study Intervention**

**Interventions, Administration, and Duration**

The short-chain fatty acid butyrate is available as a supplement in the form of capsules. One capsule (BioCare® Butyric Acid; Biocare Ltd, Birmingham, UK) contains 605 mg of butyric acid. Targeted dose of butyrate is 100 mg/kg/day, divided in two daily doses, for 3 weeks.

**Sample Collection and Analyses**

Blood tests will include plasma levels of sodium, chloride, and potassium, venous blood gas analysis, blood count, creatinine, cystatin C, urea, and C-reactive protein (CRP). Urine level of chloride, glomerular filtration rate (GFR), and fecal calprotectin will be measured. All these measurements will be performed by the laboratories of the university hospitals. Serum samples will be stored for later analyses of inflammatory markers. Buccal swabs will be collected for the extraction of genomic DNA.

After home-based fecal sample collection, the samples will be immediately put in the freezer (-20°C) and thereafter, will be transferred into our research laboratory and stored long-term at -70°C. Fecal samples will be prepared for DNA extraction and microbiota analyses. Moreover, we will perform analyses for water content, total protein, intestinal alkaline phosphatase (IAP) activity, total immunoglobulins, and methylglyoxal-derived hydroimidazolone-1 (MG-H1). In-house samples or previous data from IBD subjects and healthy controls will be utilized as control populations in the analyses.

**VI Study Procedures**

**Schedule of Evaluation for Patient Population**

The study will be conducted in 2018 under supervision by the Children´s Hospital.

All participants will be examined at the study entry (time point 0, baseline). Data on participants’ health, intestinal symptoms, and diet will be recorded, and quality of life assessed based on the protocol as described previously ^10^. Their blood, urine and fecal samples will be collected. Thereafter, the patients will provide fecal samples once a week after 1, 2, and 3 weeks (time points 1, 2, and 3).

After the 3-week follow-up of all patients, patients can participate in a voluntary trial with oral butyrate. The subjects attending the butyrate trial will be followed up for another 3 weeks and they will collect fecal samples once a week at 4, 5, and 6 weeks from the baseline visit. Data on participants’ health and intestinal symptoms will be recorded during and after the butyrate trial. After the butyrate trial, blood and urine samples will be collected.

**Study Visit**

The participants will be met by a clinician for physical examination at the study entry (time point 0, baseline). The practical aspects and risks of the study will be discussed, oral and written information given, and any questions related to the study design and sampling answered. Demographic data will be extracted from the electronic medical records.

**Questionnaires**

Before the study entry, all participants will complete an internet-based survey (or a paper form) describing their overall health and medication, antibiotic use within the past 6 months, probiotic use, alcohol drinking, smoking habits, travel history, household pet ownership, intestinal symptoms, stool frequency, basic data on their diet, and quality of life. Moreover, their physical, social, emotional, and overall quality of life will be measured with a visual analog scale (VAS, from 1 to 7) by questionnaires as described ^10^. During the 3-week follow-up, and during and after the butyrate trial, the patients will fill once a week another internet-based questionnaire describing their intestinal symptoms, salt substitution, use of butyrate, and any changes in their health or well-being during the study.

**Dietary Data Collection**

The patients will keep a food record for 3 subsequent days before the first scheduled appointment at the study entry. A trained nurse will check the food records upon returning for possible omissions and controversies with the help of a picture booklet. A dietitian will record the food records with a software (AivoDiet, Aivo Finland Oy, Turku, Finland) that utilizes a national database of foods. The use of dietary supplements will be queried in the food records and their compositions will be checked from the manufacturers.

**VII Risks and assessments**

**Risks**

Butyrate supplement is considered safe, is freely available, and approved as a supplement. This compound involves no safety issues or side effects that might cause significant risks for the participants. In terms of laboratory testing, there are no risks or harms other than sampling and data might also be relevant for the follow-up and treatment of CLD.

**Benefits**

It is possible that attending this study will not be of direct benefit for a single participant. However, the results of the project may influence the development of new treatments and follow-up recommendations that benefit the entire population of patients with CLD.

**VIII Statistical Considerations**

**Sample Size**

The sample size will be limited for the rarity of CLD. This might cause bias in analyses and small but real differences in the gut microbiota or effects of butyrate may not be detectable.

**Data Analyses**

Data analyses will be carried out by following the clinical standards of the participating hospitals. For microbiota and fecal biomarker analyses, state-of-the-art methods will be utilized in the research laboratory of the study team.

**IX Data Collection**

**Data Collection and Management**

Because of the limited number of participants, the same study nurse and clinician will meet all the participants.

**Quality Assurance**

All participants will be met by the same personnel and the laboratory analyses will be performed either in the laboratories of the university hospitals or of the study team. This ensures following the same protocols and similar handling for all samples and data.

**X Participant Rights and Confidentiality**

**Institutional Review Board Review**

Study protocol has been approved by the ethics committee of the Hospital District of Helsinki and Uusimaa (HUS/895/2017).

**Participant Confidentiality and Security**

Information about the sample and the donor is processed only by the research personnel in accordance with the data security requirements for confidential data. Data protection is secured by encoding the samples, which means that your information or any personal data cannot be detected from the results of this study or from the reports or publications. Therefore, individual results from this research project will not be reported to participants. The results from this research project will be published in peer-reviewed international journals.

**Study Discontinuation**

Participants have the right to withdraw their consent at any time without giving a specific explanation. If they withdraw the consent, their samples and personal data or other data will no longer be used in the research project. Refusing consent or withdrawing it later does not affect the availability of healthcare services.

**References**

1. Hoglund P, Haila S, Socha J, et al. Mutations of the Down-regulated in adenoma (DRA) gene cause congenital chloride diarrhoea. Nat Genet 1996;14:316-319.

2. Wedenoja S, Pekansaari E, Hoglund P, Makela S, Holmberg C, Kere J. Update on SLC26A3 mutations in congenital chloride diarrhea. Hum Mutat 2011;32:715-722.

3. Moseley RH, Hoglund P, Wu GD, et al. Downregulated in adenoma gene encodes a chloride transporter defective in congenital chloride diarrhea. Am J Physiol 1999;276:G185-92.

4. Hihnala S, Hoglund P, Lammi L, Kokkonen J, Ormala T, Holmberg C. Long-term clinical outcome in patients with congenital chloride diarrhea. J Pediatr Gastroenterol Nutr 2006;42:369-375.

5. Wedenoja S, Ormala T, Berg UB, et al. The impact of sodium chloride and volume depletion in the chronic kidney disease of congenital chloride diarrhea. Kidney Int 2008;74:1085-1093.

6. Topping DL, Clifton PM. Short-chain fatty acids and human colonic function: roles of resistant starch and nonstarch polysaccharides. Physiol Rev 2001;81:1031-1064.

7. Canani RB, Terrin G, Cirillo P, et al. Butyrate as an effective treatment of congenital chloride diarrhea. Gastroenterology 2004;127:630-634.

8. Wedenoja S, Holmberg C, Hoglund P. Oral butyrate in treatment of congenital chloride diarrhea. Am J Gastroenterol 2008;103:252-254.

9. Canani RB, Terrin G, Elce A, et al. Genotype-dependency of butyrate efficacy in children with congenital chloride diarrhea. Orphanet J Rare Dis 2013;8:194-1172-8-194.

10. Turunen P, Ashorn M, Auvinen A, Iltanen S, Huhtala H, Kolho KL. Long-term health outcomes in pediatric inflammatory bowel disease: a population-based study. Inflamm Bowel Dis 2009;15:56-62.
