# Supplementary material for: Fecal microbiota in congenital chloride diarrhea and inflammatory bowel disease
Source: PLoS One. 2022 Jun 9;17(6):e0269561. doi: 10.1371/journal.pone.0269561 (PMC9182261; doi:10.1371/journal.pone.0269561)
Supplement: S1 Methods — (PDF) [file pone.0269561.s002.pdf]

Supplementary Methods

Study Visit and Data Collection

The participants were met by one of the clinicians (SL) for physical examination at the study entry (time point 0, baseline). The practical aspects and risks of the study were discussed, oral and written information given, and any questions related to the study design and sampling answered (SL and AN). Demographic data were extracted from the electronic medical records. Before the study entry, all participants completed an internet-based survey (or a paper form) describing their overall health and medication, antibiotic use within the past 6 months, probiotic use, alcohol drinking, smoking habits, travel history, household pet ownership, intestinal symptoms, stool frequency, basic data on their diet, and quality of life. Moreover, their physical, social, emotional, and overall quality of life were measured with a visual analog scale (VAS, from 1 to 7) by questionnaires as described [33]. During the 3-week follow-up, and during and after the butyrate trial, the patients filled once a week another internet-based questionnaire describing their intestinal symptoms, salt substitution, use of butyrate, and any changes in their health or well-being during the study.

Dietary Data Collection

The patients kept a food record for 3 subsequent days before the first scheduled appointment at the study entry. A trained nurse checked the food records upon returning for possible omissions and controversies with the help of a picture booklet. A dietitian recorded the food records with a software (AivoDiet, Aivo Finland Oy, Turku, Finland) that utilizes a national database of foods. The use of dietary supplements was

also queried in the food records and their compositions were checked from the manufacturers.

Fecal Biomarker Analyses

For fecal biomarker analyses, fecal samples (50 mg of wet mass) were homogenized in 500 µL extraction buffer (10 mM Tris-HCl pH 8.0, 1 mM $MgCl_2$, 0.1 mM $ZnCl_2$,) using 0.1 mm glass beads (Precellys, Bertin Technologies, Montigny, France) following centrifugation at 13,000xg for 10min at +4°C. Supernatant was transferred into a new tube and EDTA free protease inhibitor cocktail was added to the sample (1:50) following storage at -80°C until further assay. Fecal total protein concentrations were determined with the detergent compatible DC^{TM} protein assay according to the manufacturer's instructions (Bio-Rad Laboratories, Inc, Hercules, USA).

Fecal intestinal alkaline phosphatase (IAP) activity was measured with an in-house colorimetric assay as described earlier [45]. In brief, p-nitrophenyl phosphate (pNPP) and a fixed amount of calf intestinal alkaline phosphatase was used to prepare standard curve as per the instructions (Sigma-Aldrich, St. Louis, MO, USA). Sample reaction (100 µL) contained 10 µL fecal sample, 45 µL assay buffer, 45 µL pNPP stock (4.56 mM/L) followed by incubation at 37°C for 30 min. Reaction was stopped by adding 3 M NaOH and absorbance was taken at 405 nm with the correction wavelength set to 630 nm. Samples with higher IAP activity were diluted from 1:10 to 1:200 according to subsequent sample analysis and further normalized with total fecal protein concentrations determined by the DC^{TM} protein assay.

Fecal levels of methylglyoxal modified hydro-imidazolone (MG-H1) protein adducts were determined according to manufacturer instructions using competitive enzymelinked immunosorbent assay (ELISA) (OxiSelect Methylglyoxal Competitive

Elisa Kit, STA-811-5, Cell Biolabs, San Diego, USA). The samples containing higher MG-H1 levels were diluted 1:20 with 1 x PBS containing 0.1% bovine serum albumin (BSA) according to instructions followed by incubation on an orbital shaker for 10 min at room temperature. The amount of MG-H1 was determined by comparing with the known MG-BSA standard curve and results were expressed as µg/mL.

The fecal levels of total secretory IgA, IgG and IgM antibodies were determined by a chemiluminescence immunoassay as described earlier (Antioxid Redox Signal 2013; 19: 1047–62). In brief, fecal samples (0.1 g) were suspended in ice-cold PBS buffer (1 mL) containing 0.05 % NaN_3 and 0.27 mM/L EDTA followed by centrifugation at 1,500xg for 20 min at +4°C. Further, protease inhibitors (100 mM/L PMSF and Sigma FAST protease inhibitor cocktail) were mixed with the supernatant and centrifuged at 16,000xg for 10 min at +4°C. The levels of total secretory antibodies (total IgA, IgG and IgM) were determined in the supernatants by a chemiluminescence immunoassay.

For analyses of water content, an empty 1.5 mL microcentrifuge tube was weighed and 100 µL of fecal sample was added to the tube. Then the tube containing the sample was weighed to obtain initial wet weight of the fecal sample. The mouth of the tube was covered with parafilm and the tubes were placed to the heat block at 80°C for minimum four hours to up to overnight in laminar flow hood to dry wet samples. Tubes were weighed again to obtain net dry weight and the stool water content was calculated by subtracting dry weight from the initial wet weight of stool samples.
