# Supplementary figures and images for: Fecal microbiota in congenital chloride diarrhea and inflammatory bowel disease
Source: PLoS One. 2022 Jun 9;17(6):e0269561. doi: 10.1371/journal.pone.0269561 (PMC9182261; doi:10.1371/journal.pone.0269561)

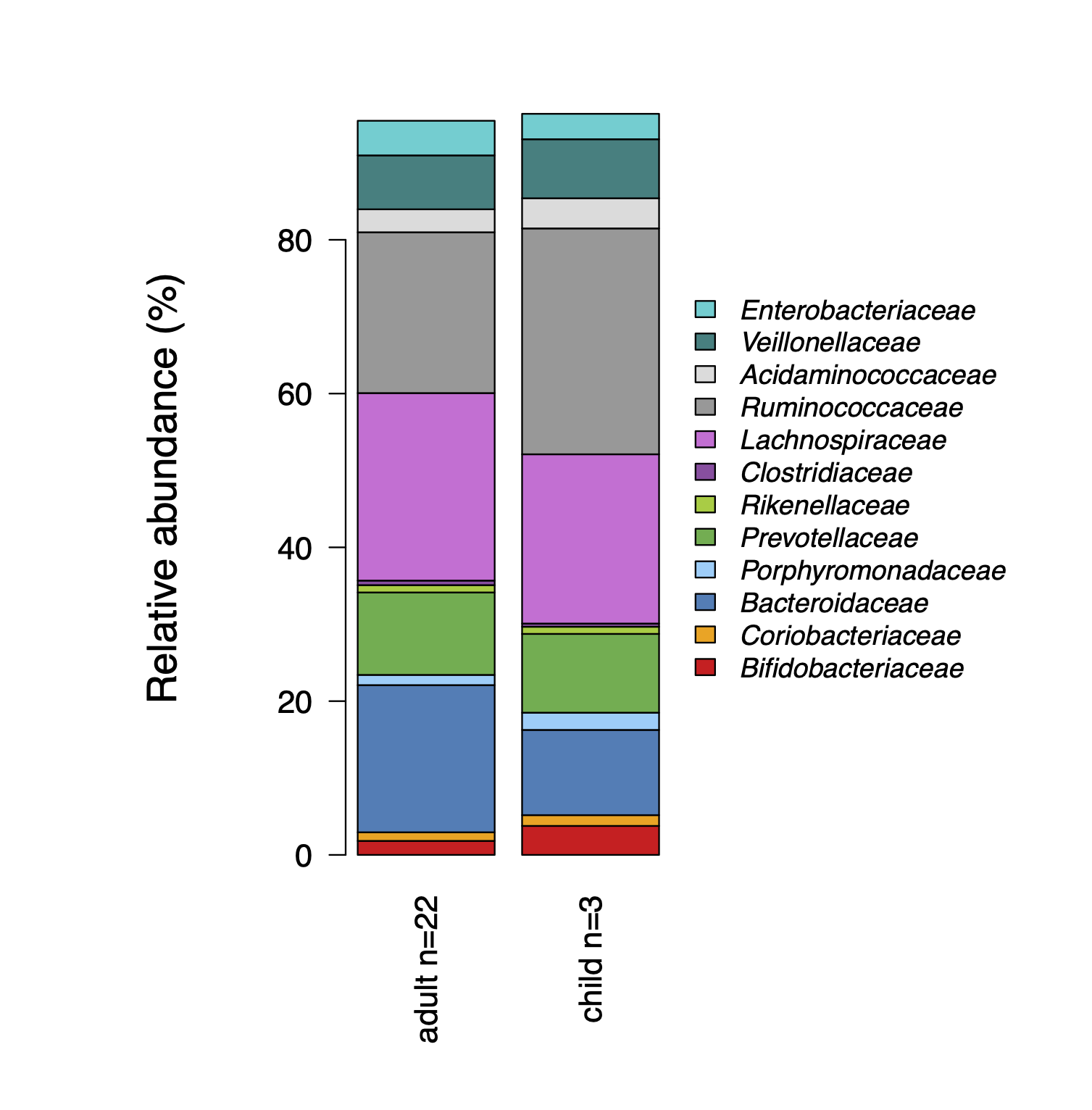

Supplement: S1 Fig — (TIFF) [file pone.0269561.s003.tiff]

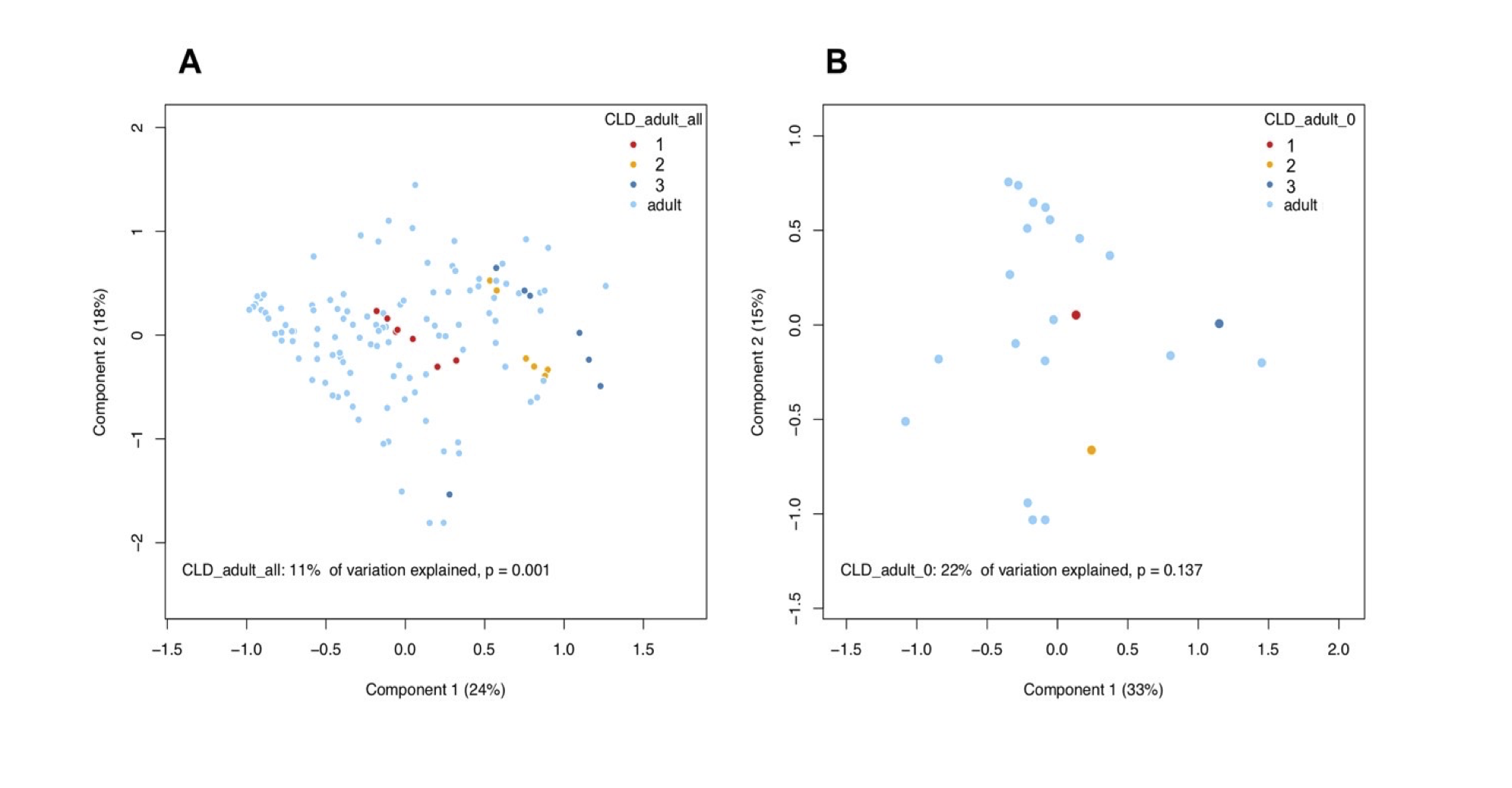

Supplement: S2 Fig — Shown are Principal-coordinated Analysis (PcoA) based on BrayCurtis dissimilarities of the samples (A) in all samples of the subjects, and (B) only in the samples taken at the study entry. The number of CLD samples in the final microbiota analyses and taken during the standard treatment were: 22 (time point 0, baseline), 23 (time point 1 week), 21 (time point 2 weeks), and 23 (time point 3 weeks). During the butyrate trial, fecal microbiota were further analyzed from 14 (time point 4 weeks), 12 (time point 5 weeks), and 14 (time point 6 weeks) samples. (TIFF) [file pone.0269561.s004.tiff]

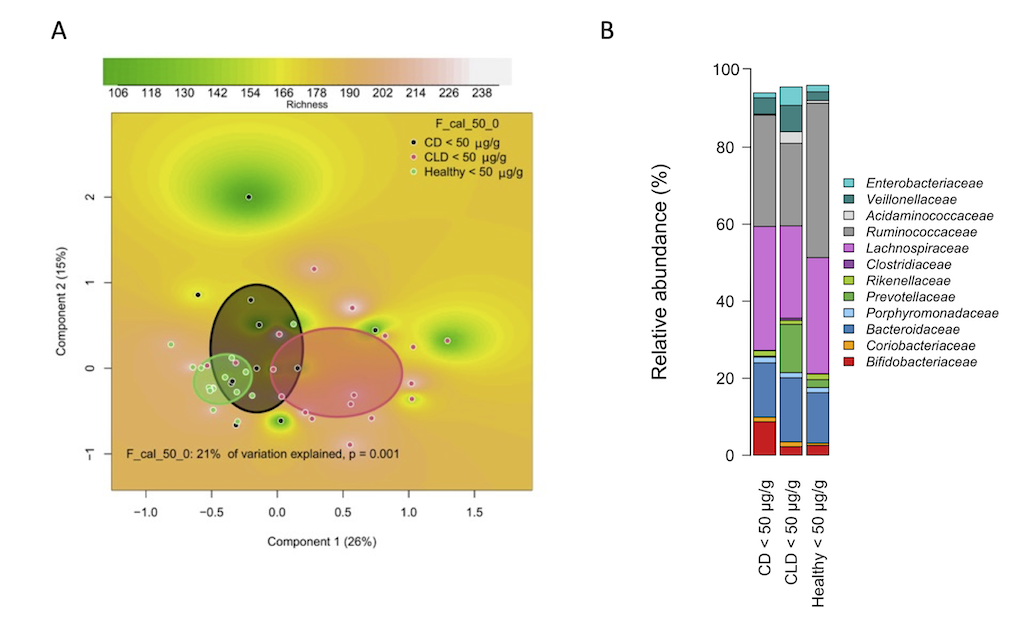

Supplement: S3 Fig — Shown are (A) Principal-coordinated Analysis (PcoA) based on Bray-Curtis dissimilarities of the richness of the microbiota, and (B) clustered stacked column graphs demonstrating microbiota differences at the genus level. The clusters in (A) are shown by circles, which were drawn based on the standard deviations of the data points in each category of the samples (CD, Crohn’s disease; CLD, Congenital chloride diarrhea; Healthy). (TIFF) [file pone.0269561.s005.tiff]

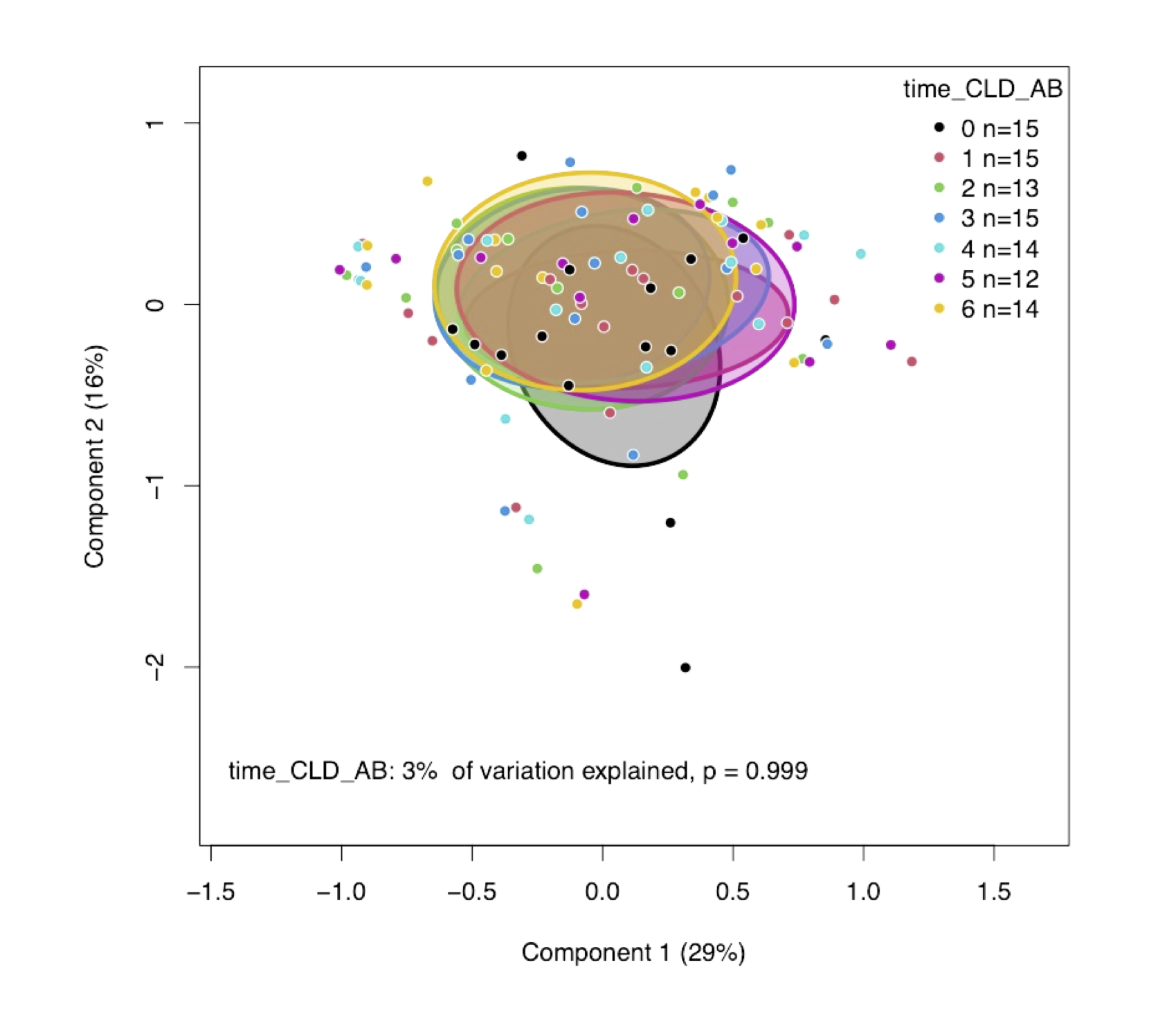

Supplement: S4 Fig — Shown are Principal-coordinated Analysis (PcoA) based on Bray-Curtis dissimilarities of the richness of the microbiota at the study entry (time point 0) and further during the standard salt substitution (time points 1, 2, and 3 weeks), and during the butyrate trial (time points 4, 5, and 6 weeks). (TIFF) [file pone.0269561.s006.tiff]

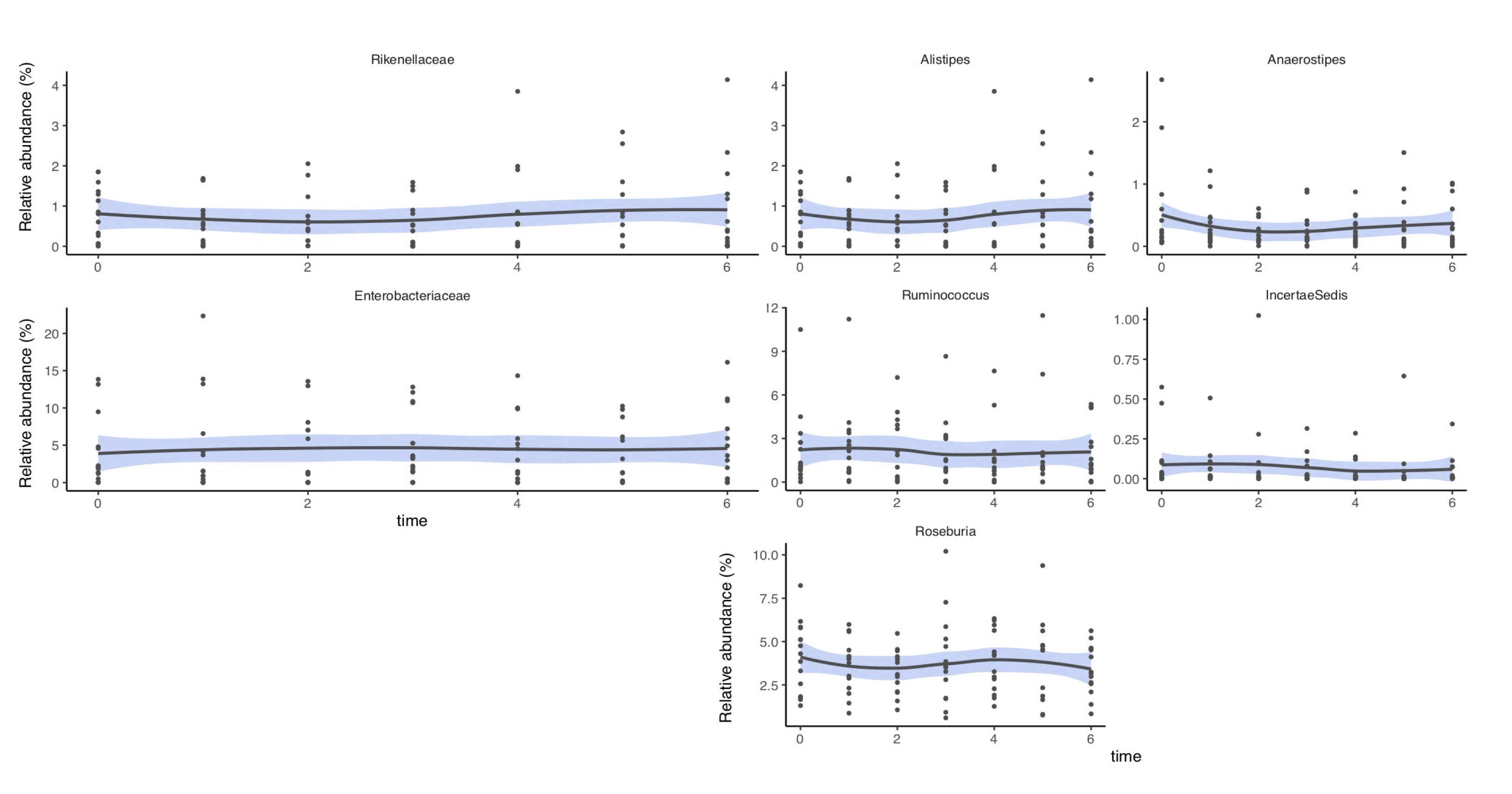

Supplement: S5 Fig — Shown are microbiota composition for the taxa with significant differences (P<0.01) (CovariateTest) between the first and last measurement (time point 3 vs 6). Similar data for Lachnospiraceae is shown in Fig 5B of the main article. Lines define group means and shaded areas 95% confidence intervals. (TIFF) [file pone.0269561.s007.tiff]

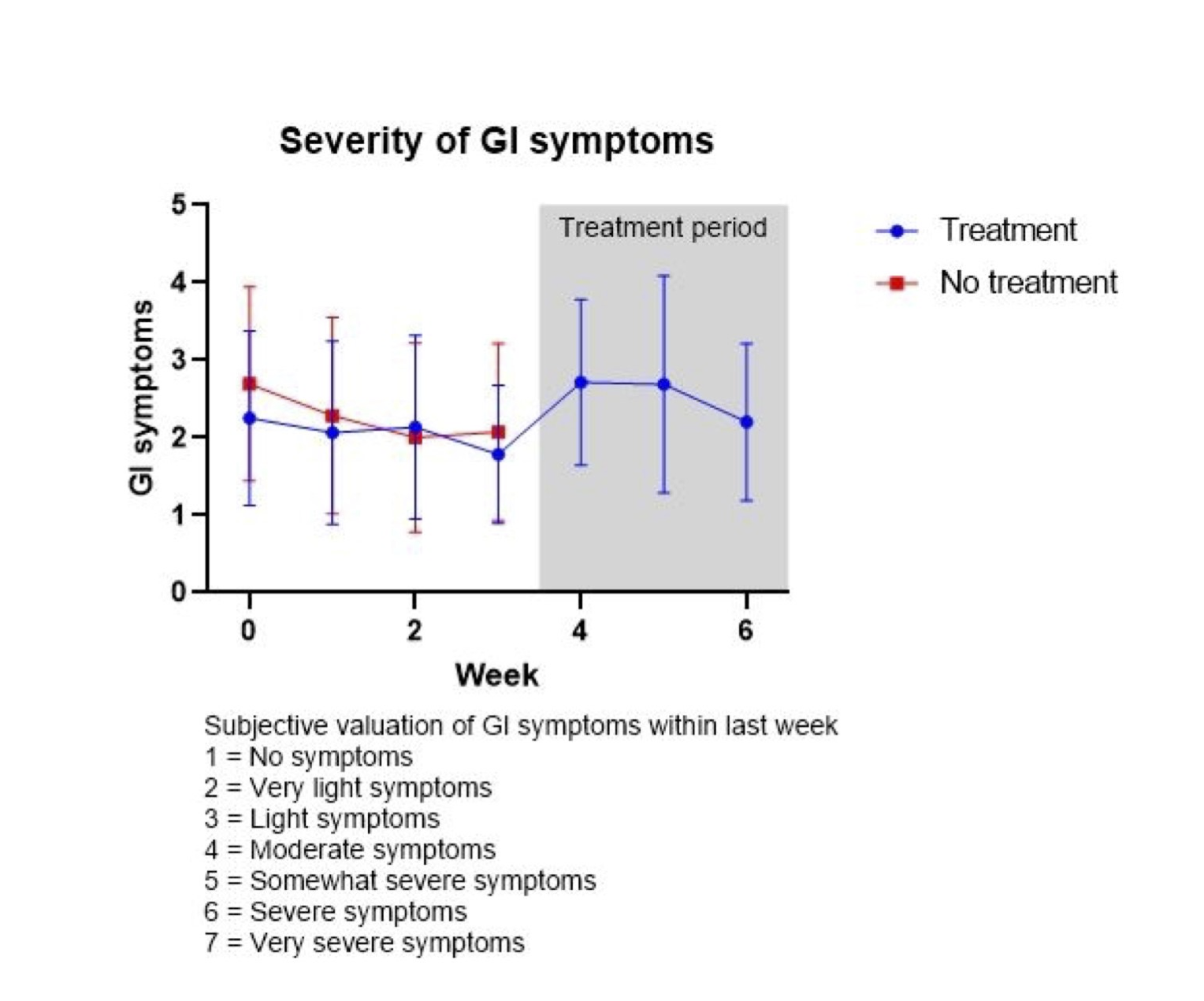

Supplement: S6 Fig — Treatment group indicates the group of subjects who attended the 3-week butyrate trial. No treatment defines the group with the standard salt substitution therapy and follow-up period of 3 weeks. Lines define the mean values and whiskers standard deviations. (TIFF) [file pone.0269561.s008.tiff]

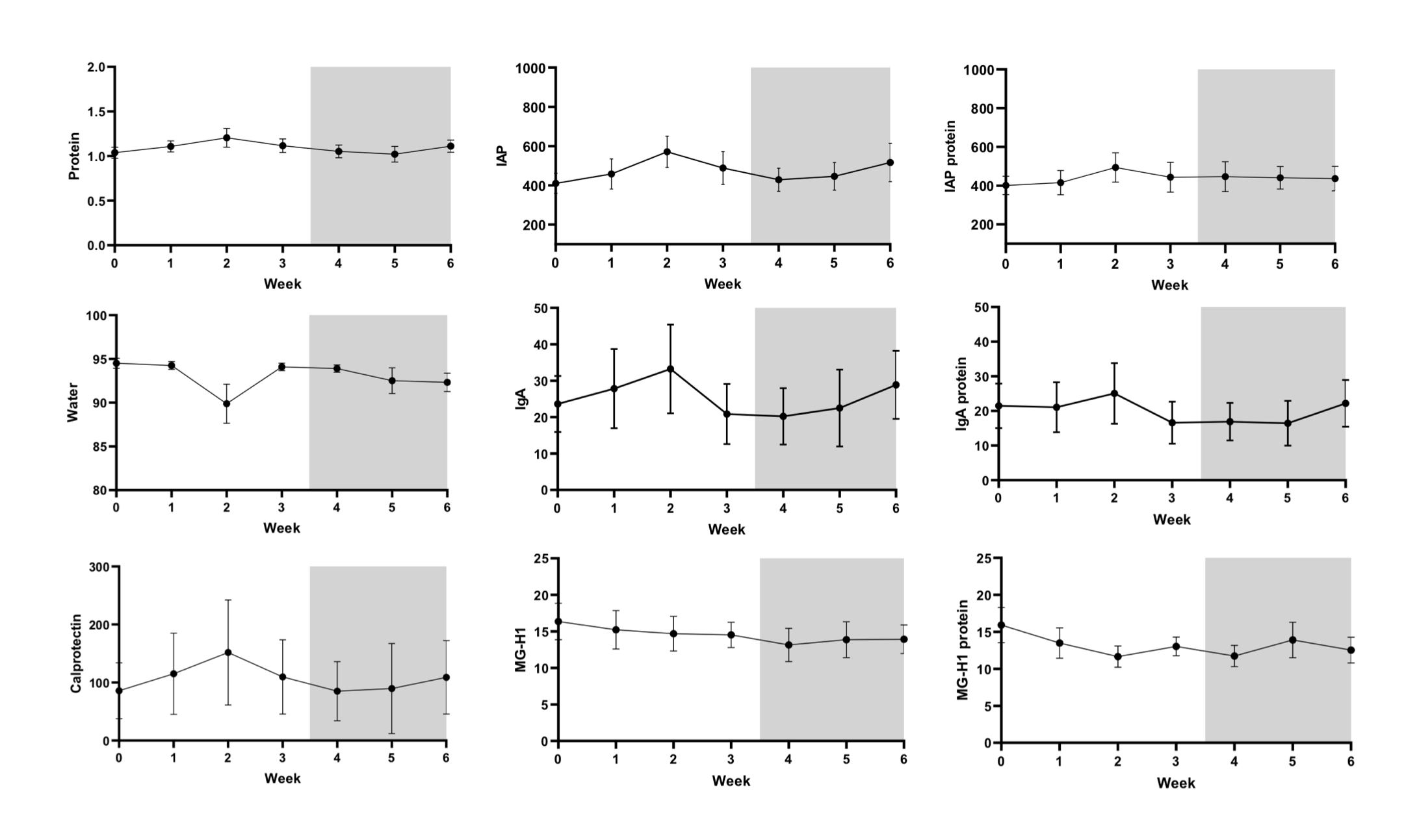

Supplement: S7 Fig — Line graphs show biomarker levels from the baseline to 6 weeks at the end of the follow-up period. Data are presented as mean with standard errors of mean. IAP, intestinal alkaline phosphatase. MG-H1, methylglyoxal-hydro-imidazolone. Shaded areas define samples taken during the butyrate treatment. (TIFF) [file pone.0269561.s009.tiff]

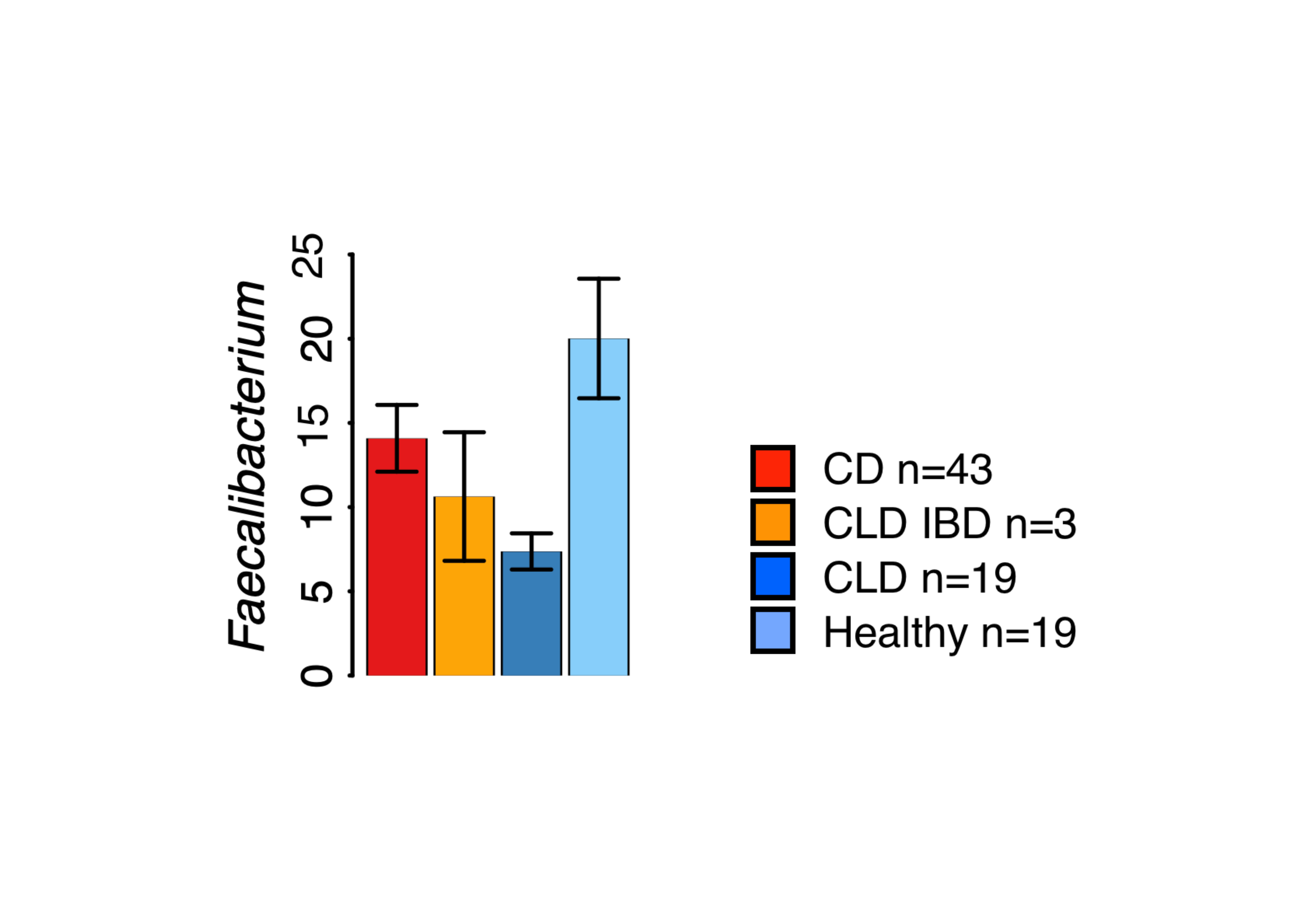

Supplement: S8 Fig — Data are presented as group means and standard errors of the mean. (TIFF) [file pone.0269561.s010.tiff]
