# Supplementary material for: Fecal microbiota in congenital chloride diarrhea and inflammatory bowel disease
Source: PLoS One. 2022 Jun 9;17(6):e0269561. doi: 10.1371/journal.pone.0269561 (PMC9182261; doi:10.1371/journal.pone.0269561)
Supplement: S1 Table — (PDF) [file pone.0269561.s011.pdf]

Number of subjects	30	
Male	13 (43%)	
Female	17 (57%)	
Age (years)	31	16-37
Height (cm)	165	160-176
Weight (kg)	68	53-80
BMI (kg/m^2)	23.3	20.1-26.5
Chloride substitution (mmol/kg/day)	3.0	2.4-3.6
Fecal calprotectin (ug/g)	9.0	2.5-32.8
Hemoglobin (g/L)	140	132-153
P-CRP (mg/L)	1.5	1.5-10.0
vB-pH	7.37	7.34-7.42
vB-BE (mmol/L)	3.1	0.8-5.0
vB-HCO3 (mmol/L)	27	24-29
P-K (mmol/L)	4.0	3.7-4.3
P-Na (mmol/L)	139	138-140
P-Cl (mmol/L)	104	100-106
P-Creatinine (µmol/L)	65	58-77
eGFR (mL/min/1.73m^2)	102	89.0-112
P-Ca-Ion (mmol/L)	1.2	1.18-1.23
P-Cystatin C (mg/L)	0.92	0.89-1.03
P-Urea (mmol/L)	5.4	4.2-6.3
U-Cl (mmol/L)	54	13-110
Bristol Stool Scale (BSS)	7*	

Values are expressed in number (percentage) and median (interquartile range, IQR). BMI, body mass index. P, plasma. CRP, C-reactive protein. vB, venous blood. BE, base excess. HCO3, bicarbonate. eGFR, estimated glomelural filtration rate. U, urine. Bristol stool scale (BSS): type 1–2 indicates constipation, 3–4 normal, and 5–7 diarrhea. *all patients reported the BSS of 7.
