# Supplementary material for: Fecal microbiota in congenital chloride diarrhea and inflammatory bowel disease
Source: PLoS One. 2022 Jun 9;17(6):e0269561. doi: 10.1371/journal.pone.0269561 (PMC9182261; doi:10.1371/journal.pone.0269561)
Supplement: S2 Table — (PDF) [file pone.0269561.s012.pdf]

	CLD (n=22)	CD (n=43)	Healthy (n=19)	*P* value
Mean richness	186[a,b]	151[a,c]	211[b,c]	[a] **3.08e-05**<br>[b] 0.58<br>[c] **0.0002**
Mean diversity	26.2[a,b]	15.2[a,c]	19.7[b,c]	[a] **3.34e-08**<br>[b] **0.004**<br>[c] **0.005**

Pairwise comparisons (Student t-test) were performed between two subgroups as indicated (a,b,c). CLD (congenital chloride diarrhea) samples were taken at the study entry from 22 adults/adolescents, whose samples were included in the final microbiota analyses.
