# Supplementary material for: Fecal microbiota in congenital chloride diarrhea and inflammatory bowel disease
Source: PLoS One. 2022 Jun 9;17(6):e0269561. doi: 10.1371/journal.pone.0269561 (PMC9182261; doi:10.1371/journal.pone.0269561)
Supplement: S3 Table — P values for microbiota differences in congenital chloride diarrhea (CLD), CLD-associated IBD (CLD IBD), and Crohn’s disease (CD), in comparison with the microbiota of healthy controls (n = 19). Shown are only the taxa with adjusted P values (FDR_p) <0.1 after comparison of the subgroup (CD, CLD IBD, or CLD) and healthy controls (GroupTest). (PDF) [file pone.0269561.s013.pdf]

taxon	CD n=43_p	CLD IBD n=3_p	CLD n=19_p	CD n=43_p_FDR	CLD IBD n=3_p_FDR	CLD n=19_p_FDR	FoldChange_ CD n=43	FoldChange_ CLD IBD n=3	FoldChange_ CLD n=19
Actinobacteria_Coriobacteriia	0.11978771260861	0.854522715654807	**0.00316570116228702**	0.303462205275145	0.959334958678074	**0.00859261744049333**	1.41170607064691	0.912666750260927	2.16043968311085
Actinobacteria_Coriobacteriia_Coriobacteriales	0.11978771260861	0.854522715654807	**0.00316570116228702**	0.303462205275145	0.959334958678074	**0.00859261744049333**	1.41170607064691	0.912666750260927	2.16043968311085
Actinobacteria_Coriobacteriia_Coriobacteriales_Coriobacteriaceae	0.11978771260861	0.854522715654807	**0.00316570116228702**	0.303462205275145	0.959334958678074	**0.00859261744049333**	1.41170607064691	0.912666750260927	2.16043968311085
Bacteroidetes_Bacteroidia_Bacteroidales_Prevotellaceae_Prevotella	0.569209204013451	**5.22123058857017e-06**	**1.94784796752571e-05**	0.809311149177995	**6.61355874552222e-05**	**0.000148036445531954**	0.390315580871415	3.54514977433556	5.08124680605559
Bacteroidetes_Bacteroidia_Bacteroidales_Prevotellaceae_uncultured	0.283705571108919	0.584047951249667	**0.00545129897429045**	0.554214995199379	0.792636505267405	**0.0122196639568492**	0.632392051830751	0.835061663496337	6.00433176153574
Bacteroidetes_Bacteroidia_Bacteroidales_Rikenellaceae	0.000969382126197908	0.314067901009859	0.0313562759200886	0.0163677965088785	0.54248091992612	0.0567399278553984	0.716930098763832	0.539596316592831	0.639017221358286
Bacteroidetes_Bacteroidia_Bacteroidales_Rikenellaceae_Alistipes	0.000969382126197908	0.314067901009859	0.0313562759200886	0.0163677965088785	0.54248091992612	0.0567399278553984	0.716930098763832	0.539596316592831	0.639017221358286
Firmicutes_Clostridia_Clostridiales_Christensenellaceae	0.00577403590917813	0.0804153714183019	0.183346921033033	0.0313447663641098	0.254648676157956	0.248827964259116	0.360761341120226	0.232444888385863	0.560230570934139
Firmicutes_Clostridia_Clostridiales_Christensenellaceae_uncultured	0.00577403590917813	0.0804153714183019	0.183346921033033	0.0313447663641098	0.254648676157956	0.248827964259116	0.360761341120226	0.232444888385863	0.560230570934139
Firmicutes_Clostridia_Clostridiales_Lachnospiraceae	0.0239102281706811	**2.82155840486157e-05**	0.028171113275889	0.100954296720654	**0.000268048048461849**	0.0563422265517781	1.17575795165947	0.755128262259807	0.839863800904294
Firmicutes_Clostridia_Clostridiales_Lachnospiraceae_Blautia	0.00129219446122725	**0.00114472643423236**	0.413361192296268	0.0163677965088785	**0.00869992090016597**	0.490866415851818	2.14818169376526	0.500837893745576	0.839551585190568
Firmicutes_Clostridia_Clostridiales_Lachnospiraceae_Pseudobutyrivibrio	0.649691171784829	0.212141410246123	0.03376797527504	0.851319466476672	0.424282820492245	0.0583265027477964	0.863036994569628	0.401313549342535	0.444289803533082
Firmicutes_Clostridia_Clostridiales_Lachnospiraceae_Roseburia	0.0178922212408898	0.523027282189136	0.690062331473585	0.0849880508942266	0.736112471229154	0.690062331473585	0.667474011037554	0.781886294569811	1.08358139826918
Firmicutes_Negativicutes_Selenomonadales_Acidaminococcaceae_Acidaminococcus	0.818366319214966	0.0374145979389637	**0.00997558177016529**	0.957189022904766	0.142175472168062	**0.0210595615147934**	0.487545693975804	1.33145515878263	3.73009650342022
Firmicutes_Negativicutes_Selenomonadales_Veillonellaceae_Dialister	0.00318479878913002	0.269984414646527	0.0666059749820347	0.0242044707973881	0.512970387828401	0.110044654318144	0.771755161372958	1.74220926136316	0.666410636034042
Firmicutes_Negativicutes_Selenomonadales_Veillonellaceae_Veillonella	0.00180088659232714	**0.00341002299289449**	**2.33885554072877e-06**	0.0171084226271078	**0.0161976092162488**	**2.96255035158977e-05**	5.28896913241367	11.6108763369638	9.94601139580372
Fusobacteria	0.931999838091483	0.136632900835308	**0.00022280601933503**	0.957189022904766	0.305414719514218	**0.000769693521339195**	1.04974067322089	2536.38507677476	4486.76529060896
Fusobacteria_Fusobacteriia	0.931999838091483	0.136632900835308	**0.00022280601933503**	0.957189022904766	0.305414719514218	**0.000769693521339195**	1.04974067322089	2536.38507677476	4486.76529060896
Fusobacteria_Fusobacteriia_Fusobacteriales	0.931999838091483	0.136632900835308	**0.00022280601933503**	0.957189022904766	0.305414719514218	**0.000769693521339195**	1.04974067322089	2536.38507677476	4486.76529060896
Fusobacteria_Fusobacteriia_Fusobacteriales_Fusobacteriaceae	0.931999838091483	0.136632900835308	**0.00022280601933503**	0.957189022904766	0.305414719514218	**0.000769693521339195**	1.04974067322089	2536.38507677476	4486.76529060896
Fusobacteria_Fusobacteriia_Fusobacteriales_Fusobacteriaceae_Fusobacterium	0.931999838091483	0.136632900835308	**0.00022280601933503**	0.957189022904766	0.305414719514218	**0.000769693521339195**	1.04974067322089	2536.38507677476	4486.76529060896
Proteobacteria	0.839163481177981	**2.06147039567827e-06**	**1.64763324642369e-07**	0.957189022904766	**3.91679375178872e-05**	**3.13050316820501e-06**	1.08813526666306	1.53037654292995	3.37020151010973
Proteobacteria_Betaproteobacteria	0.534985682836798	0.908843645063438	**0.00387445858824759**	0.809311149177995	0.959334958678074	**0.00981529509022722**	1.544288609614	1.09914716288809	3.54631992789487
Proteobacteria_Betaproteobacteria_Burkholderiales	0.549802411039877	0.907145196163067	**0.0054666917701694**	0.809311149177995	0.959334958678074	**0.0122196639568492**	1.53845601230324	1.10085559200831	3.36484233565979
Proteobacteria_Gammaproteobacteria	0.174888121446133	**0.00338615214929021**	**1.11907366197633e-05**	0.390926389114887	**0.0161976092162488**	**0.000106311997887752**	1.07656217371886	1.57747231747022	3.46551866536026
Proteobacteria_Gammaproteobacteria_Enterobacteriales_Enterobacteriaceae_Citrobacter	0.226991655756171	**0.00201949374727153**	**7.95820254176707e-05**	0.479204606596361	**0.012790127066053**	**0.000504019494311915**	0.457493215156388	2.15425044871814	3.53275050206963
Proteobacteria_Gammaproteobacteria_Enterobacteriales_Enterobacteriaceae_Escherichia	0.0556781701512876	**3.33998968693929e-13**	**2.49513961643892e-09**	0.176314205479077	**1.26919608103693e-11**	**9.48153054246788e-08**	15.4752106997209	12.7167093881759	45.7324484818413

*For technical reason, no exact P value could be calculated for Ruminococcaceae. **4 outliers (all non-IBD subjects) in CLD group for the Fusobacteria explain the high fold change p, P value. p_FDR, adjusted P value after Benjamini-Hochberg correction.
