# Supplementary material for: Fecal microbiota in congenital chloride diarrhea and inflammatory bowel disease
Source: PLoS One. 2022 Jun 9;17(6):e0269561. doi: 10.1371/journal.pone.0269561 (PMC9182261; doi:10.1371/journal.pone.0269561)
Supplement: S4 Table — Daily intakes of energy and energy-yielding nutrients in patients with congenital chloride diarrhea (CLD; n = 30). Values outside the reference range are shown bolded. (PDF) [file pone.0269561.s014.pdf]

	**Median (IQR) intake**	**Recommendation**
Energy (kcal)	1959 (1572-2550)	
Protein (g)	77.0 (56.9-106.5)	
Protein (E%)	16.0 (13.9-18.1)	10-20
Carbohydrates (g)	205.3 (170.2-250.7)	
**Carbohydrates (E%)**	**42.3 (35.2-47.4)**	45-60
Starch (g)	93.9 (75.6-116.0)	
Sucrose (g)	54.0 (37.0-74.1)	
**Sucrose (E%)**	**11.7 (7.9-15.2)**	<10
Lactose (g)	10.7 (5.7-24.9)	
Fructose (g)	12.7 (6.8-19.4)	
Fat (g)	81.8 (60.7-108.4)	
Fat (E%)	38.4 (33.2-41.7)	25-40
SAFA (g)	30.9 (21.4-39.6)	
**SAFA (E%)**	**14.0 (11.6-16.6)**	<10
MUFA (g)	27.2 (21.2-38.5)	
MUFA (E%)	13.3 (10.3-15.19)	10-20
PUFA (g)	12.7 (9.7-16.7)	
PUFA (E%)	6.0 (4.4-6.9)	5-10
**Fiber (g)**	**14.9 (10.9-19.0)**	25-35

E%, percentage of energy; SAFA, saturated fatty acids; MUFA, monounsaturated fatty acids; PUFA, polyunsaturated fatty acids.
