# Supplementary material for: Fecal microbiota in congenital chloride diarrhea and inflammatory bowel disease
Source: PLoS One. 2022 Jun 9;17(6):e0269561. doi: 10.1371/journal.pone.0269561 (PMC9182261; doi:10.1371/journal.pone.0269561)
Supplement: S5 Table — Daily intakes of vitamins, minerals and trace elements in patients with congenital chloride diarrhea (CLD; n = 30). Values outside the reference range are shown bolded. (PDF) [file pone.0269561.s015.pdf]

	Median (IQR) intake	Median (IQR) intake, percentage of recommendation
Vitamin A (µg)	803.6 (544.7-1070.4)	110.6 (72.5-146.4)
**Vitamin D diet (µg)**	**7.1 (3.7-10.4)**	**71.0 (36.8-103.9)**
Vitamin D total (µg)	12.4 (6.4-19.1)	124.0 (64.4-190.5)
Vitamin E diet (mg)	9.8 (7.3-15.1)	109.3 (91.5-167.4)
Vitamin E total (mg)	9.8 (7.7-15.1)	119.1 (92.8-168.2)
Thiamin diet (mg)	1.2 (0.9-1.5)	109.7 (85.2-133.3)
Thiamin total (mg)	1.2 (1.0-1.7)	111.9 (88.0-143.1)
Riboflavin diet (mg)	1.7 (1.2-2.6)	136.0 (99.1-198.4)
Riboflavin total (mg)	1.9 (1.4-2.8)	144.9 (99.1-207.3)
Niacin diet (mg)	33.1 (22.6-44.2)	222.3 (168.1-259.7)
Niacin total (mg)	33.4 (22.6-51.9)	232.4 (168.1-294.7)
Pyridoxin diet (mg)	2.0 (1.3-2.6)	165.9 (124.3-196.6)
Pyridoxin total (mg)	2.0 (1.6-2.9)	168.1 (135.5-201.7)
**Folate diet (µg)**	**200.7 (171.9-287.2)**	**78.7 (61.0-122.1)**
**Folate total (µg)**	**202.2 (174.7-319.4)**	**82.7 (61.0-141.0)**
Vitamin B12 diet (µg)	5.2 (3.8-9.9)	282.2 (193.9-493.0)
Vitamin B12 total (µg)	5.5 (3.8-10.9)	290.1 (193.9-546.9)
**Vitamin C diet (mg)**	**84.0 (42.0-147.0)**	**85.1 (42.6-147.0)**
Vitamin C total (mg)	92.3 (42.6-180.0)	123.1 (56.7-213.1)
Iron (mg)	10.3 (8.5-16.7)	95.8 (64.0-148.2)
Calcium diet (mg)	998.7 (724.5-1353.1)	130.6 (90.6-165.2)
Calcium total (mg)	1022.2 (729.8-1353.1)	132.2 (91.2-165.2)
Magnesium diet (mg)	282.2 (219.1-404.6)	110.1 (79.3-135.1)
Magnesium total (mg)	296.4 (219.1-408.0)	118.4 (79.3-140.8)
Sodium diet (mg)	3219.1 (2170.5-3842.7)	N.A.
Potassium diet (mg)	2891.7 (2557.1-4344.7)	108.6 (83.3-135.5)
Phosphorus (mg)	1340.8 (1022.6-1786.1)	209.3 (177.0-297.7)
Selenium diet (µg)	66.3 (44.6-89.3)	130.0 (98.4-174.6)
Selenium total (µg)	66.3 (44.6-91.7)	130.0 (98.4-181.4)
Zinc diet (mg)	10.8 (7.9-15.3)	137.0 (101.4-197.8)
Zinc total (mg)	11.6 (7.9-17.3)	141.0 (101.4-206.5)
Copper (µg)	1.09 (0.87-1.43)	138.9 (110.8-180.1)

IQR, interquartile range.
