# Supplementary material for: Fecal microbiota in congenital chloride diarrhea and inflammatory bowel disease
Source: PLoS One. 2022 Jun 9;17(6):e0269561. doi: 10.1371/journal.pone.0269561 (PMC9182261; doi:10.1371/journal.pone.0269561)
Supplement: S6 Table — P values for fecal microbiota composition in relation to dietary lactose (grams/day) in congenital chloride diarrhea (CLD; n = 22) and healthy controls (n = 19). Shown are only the taxa with adjusted P values (FDR) <0.1 (CovariateTest). p, P value. FDR, adjusted P value after Benjamini-Hochberg correction. (PDF) [file pone.0269561.s016.pdf]

taxon	LACS_f_CLD n=22_p	LACS_f_Healthy n=19_p	LACS_f_CLD n=22_FDR	LACS_f_Healthy n=19_FDR
Actinobacteria_Coriobacteriia_Coriobacteriales_Coriobacteriaceae_Slackia	0.691827128851914	2.2621471827834e-28	0.822539511979127	4.37348455338123e-27
Bacteroidetes	0.548568550444128	0.000168561297656562	0.822539511979127	0.00108628391823118
Bacteroidetes_Bacteroidia	0.548568550444128	0.000168561297656562	0.822539511979127	0.00108628391823118
Bacteroidetes_Bacteroidia_Bacteroidales	0.548568550444128	0.000168561297656562	0.822539511979127	0.00108628391823118
Bacteroidetes_Bacteroidia_Bacteroidales_Porphyromonadaceae	NA	0.0019373551762476	NA	0.00864358463248931
Bacteroidetes_Bacteroidia_Bacteroidales_Prevotellaceae	NA	3.01526104090972e-53	NA	1.74885140372764e-51
Bacteroidetes_Bacteroidia_Bacteroidales_Prevotellaceae_Prevotella	NA	3.31601854077464e-29	NA	9.61645376824646e-28
Firmicutes_Clostridia	0.0606206640283216	8.22855038260199e-08	0.646620416302097	9.54511844381831e-07
Firmicutes_Clostridia_Clostridiales	0.0606206640283216	8.22855038260199e-08	0.646620416302097	9.54511844381831e-07
Firmicutes_Clostridia_Clostridiales_Lachnospiraceae	0.876431086221528	4.14274960554326e-06	0.890342690764727	4.00465795202515e-05
Firmicutes_Clostridia_Clostridiales_Ruminococcaceae_Faecalibacterium	0.660847992196561	0.000231648298557668	0.822539511979127	0.00134356013163448
Proteobacteria_Betaproteobacteria	0.0806938838162997	0.000303566332850315	0.648799307830211	0.00146957758826096
Proteobacteria_Betaproteobacteria_Burkholderiales	0.0452826947989495	0.000304050535502267	0.646620416302097	0.00146957758826096
