# Supplementary material for: Fecal microbiota in congenital chloride diarrhea and inflammatory bowel disease
Source: PLoS One. 2022 Jun 9;17(6):e0269561. doi: 10.1371/journal.pone.0269561 (PMC9182261; doi:10.1371/journal.pone.0269561)
Supplement: S7 Table — P values for fecal microbiota composition in relation to dietary total fiber (grams/day) in congenital chloride diarrhea (CLD; n = 22) and healthy controls (n = 19). Shown are only the taxa with adjusted P values (FDR) <0.1 (CovariateTest). p, P value. FDR, adjusted P value after Benjamini-Hochberg corrections. (PDF) [file pone.0269561.s017.pdf]

taxon	FIBC_f_CLD n=22_p	FIBC_f_Healthy n=19_p	FIBC_f_CLD n=22_FDR	FIBC_f_Healthy n=19_FDR
Actinobacteria_Coriobacteriia_Coriobacteriales_Coriobacteriaceae_Slackia	0.759493400481376	2.30890249518919e-68	0.951052750852658	1.38534149711351e-66
Bacteroidetes_Bacteroidia_Bacteroidales_Bacteroidaceae	0.685377621143223	0.00179532316283044	0.951052750852658	0.0215438779539653
Bacteroidetes_Bacteroidia_Bacteroidales_Bacteroidaceae_Bacteroides	0.685377621143223	0.00179532316283044	0.951052750852658	0.0215438779539653
Proteobacteria_Gammaproteobacteria	0.870400250489035	0.0057286142734829	0.951052750852658	0.057286142734829
Proteobacteria_Gammaproteobacteria_Enterobacteriales	0.940460006118589	0.00022430687350755	0.951052750852658	0.00448613747015099
Proteobacteria_Gammaproteobacteria_Enterobacteriales_Enterobacteriaceae	0.940460006118589	0.00022430687350755	0.951052750852658	0.00448613747015099
