# Supplementary material for: Fecal microbiota in congenital chloride diarrhea and inflammatory bowel disease
Source: PLoS One. 2022 Jun 9;17(6):e0269561. doi: 10.1371/journal.pone.0269561 (PMC9182261; doi:10.1371/journal.pone.0269561)
Supplement: S8 Table — P values for fecal microbiota composition in relation to dietary sucrose (% of energy intake) in congenital chloride diarrhea (CLD; n = 22) and healthy controls (n = 19). Shown are only the taxa with adjusted P values (FDR) <0.1 (CovariateTest). p, P value. FDR, adjusted P value after Benjamini-Hochberg correction. (PDF) [file pone.0269561.s018.pdf]

taxon	SUCS_pros_CLD n=22_p	SUCS_pros_Healthy n=19_p	SUCS_pros_CLD n=22_FDR	SUCS_pros_Healthy n=19_FDR
Bacteroidetes_Bacteroidia_Bacteroidales_Prevotellaceae	0.0949858035597122	0	0.489542218346209	0
Bacteroidetes_Bacteroidia_Bacteroidales_Prevotellaceae_Prevotella	0.207855124648525	0	0.633013334156872	0
Bacteroidetes_Bacteroidia_Bacteroidales_Prevotellaceae_uncultured	0.474790607331198	1.09196418611182e-111	0.71844142976783	2.22032717842737e-110
Firmicutes_Bacilli_Lactobacillales_Streptococcaceae	0.0698323526284945	0.00416332541457972	0.465700654099338	0.0272221518154909
Firmicutes_Bacilli_Lactobacillales_Streptococcaceae_Streptococcus	0.0576189679483559	0.00101196477721	0.465700654099338	0.00771623142622622
Firmicutes_Clostridia_Clostridiales_Clostridiaceae	0.377083963046702	0.00490891262246557	0.667371102294522	0.0272221518154909
Firmicutes_Clostridia_Clostridiales_Clostridiaceae_Clostridium	0.377083963046702	0.00490891262246557	0.667371102294522	0.0272221518154909
Firmicutes_Clostridia_Clostridiales_Lachnospiraceae	0.569256610972642	0.0210424630196353	0.71844142976783	0.098737711092135
Firmicutes_Clostridia_Clostridiales_Lachnospiraceae_IncertaeSedis	**0.00151924789313595**	0.890586082012188	**0.0508948044200054**2	0.905429183379058
Firmicutes_Negativicutes_Selenomonadales_Acidaminococcaceae	NA	7.72050798883431e-24	NA	1.17737746829723e-22
Firmicutes_Negativicutes_Selenomonadales_Veillonellaceae_Veillonella	0.163883134023125	3.01907776707595e-07	0.633013334156872	3.68327487583265e-06
Proteobacteria_Betaproteobacteria	0.249319542096952	1.179172643761e-05	0.667371102294522	0.000107012635057708
Proteobacteria_Betaproteobacteria_Burkholderiales	0.228547325781747	1.22801384492452e-05	0.63802795114071	0.000107012635057708
Proteobacteria_Gammaproteobacteria_Enterobacteriales_Enterobacteriaceae_Escherichia	0.337849566981967	0.0160913788217079	0.667371102294522	0.0817978423436817
