# Supplementary material for: Fecal microbiota in congenital chloride diarrhea and inflammatory bowel disease
Source: PLoS One. 2022 Jun 9;17(6):e0269561. doi: 10.1371/journal.pone.0269561 (PMC9182261; doi:10.1371/journal.pone.0269561)
Supplement: S9 Table — P values for the changes in fecal microbiota composition during the 3-week follow-up. Data are presented separately for the group with the standard salt substitution treatment (treatment_standard) and the butyrate trial group (treatment_butyrate). For those with the standard salt substitution, P values are shown for the differences between 1, 2, and 3 weeks (p_1, p_2, and p_3) vs baseline (time point 0). For the butyrate trial group, P values are shown for the differences during the butyrate trial at 1, 2, and 3 weeks (p_1, p_2, and p_3) versus the beginning of the trial. Shown are only the taxa with adjusted P values (FDR) <0.1 after ChangeTest. p, P value. FDR, adjusted P value after Benjamini-Hochberg correction. (PDF) [file pone.0269561.s019.pdf]

taxon	p_1/treatment_standard	p_1/treatment_butyrate	p_2/treatment_standard	p_2/treatment_butyrate	p_3/treatment_standard	p_3/treatment_butyrate	p_1/treatment_standard_FDR	p_1/treatment_butyrate_FDR	p_2/treatment_standard_FDR	p_2/treatment_butyrate_FDR	p_3/treatment_standard_FDR	p_3/treatment_butyrate_FDR
Bacteroidetes_Bacteroidia_Bacteroidales_Rikenellaceae_Alistipes	0.02466830353330987	0.666641387887603	NA	NA	0.333720087218171	**7.29120601327862e-05**	0.370343759767303	0.835737493883186	NA	NA	0.915850920352629	**0.00164052135298769**
Firmicutes_Clostridia_Clostridiales_Lachnospiraceae_Anaerostipes	0.0636925568347928	0.756143446846692	**0.000237321874729157**	**0.000319348539373194**	0.160904766080474	0.635680724841165	0.668771846765324	0.835737493883186	**0.0079327352997245**	**0.00819151565063829**	0.915850920352629	0.937512059639133
Firmicutes_Clostridia_Clostridiales_Lachnospiraceae_IncertaeSedis	0.7152019756 86051	0.507292649759739	0.010820493525864	**0.00816891860766586**	0.347788027415567	0.99759960745364	0.92384345673614	0.835737493883186	0.105499811877174	**0.0796469564247421**	0.915850920352629	0.99759960745364
Firmicutes_Clostridia_Clostridiales_Lachnospiraceae_Roseburia	0.333088799496675	**0.000827786463114836**	0.0590565560812386	0.0542992713230984	0.56749903160353	0.833654673748372	0.914607808665458	**0.0173835157254116**	0.343898934695638	0.302524511657263	0.915850920352629	0.937512059639133
Firmicutes_Clostridia_Clostridiales_Lachnospiraceae_uncultured	0.2384965025 33722	0.220947696214815	**0.00040680693844741**	**0.000420077725673759**	0.137903360697296	0.145620357118045	0.914607808665458	0.583157918091933	**0.0079327352997245**	**0.00819151565063829**	0.915850920352629	0.824493707435743
Firmicutes_Clostridia_Clostridiales_Ruminococcaceae_Ruminococcus	0.423436677739196	0.222155397368356	0.0728764540658027	0.320069324959019	**0.0213152272796034**	**0.00237011774056389**	0.92384345673614	0.583157918091933	0.343898934695638	0.762583290083796	0.915850920352629	**0.0355517661084584**
Firmicutes_Negativicutes_Selenomonadales_Acidaminococcaceae_uncultured	0.671741052171222	**7.11796383842881e-11**	0.475951381527097	0.41851214190616	0.588569486602281	0.146576659099688	0.92384345673614	**2.9895448121401e-09**	0.866180395450157	0.859051238649487	0.915850920352629	0.824493707435743
Bacteroidetes_Bacteroidia_Bacteroidales_Rikenellaceae	0.02466830353330987	0.666641387887603	NA	NA	0.333720087218171	**7.29120601327862e-05**	0.370343759767303	0.835737493883186	NA	NA	0.915850920352629	**0.00164052135298769**
Firmicutes_Clostridia_Clostridiales_Lachnospiraceae	0.568971450803606	0.592176063359292	**0.00491044378156591**	**0.00778218713736731**	0.755142622908708	0.465241720221091	0.92384345673614	0.835737493883186	0.0638357691603568	**0.0796469564247421**	0.915850920352629	0.937512059639133
Firmicutes_Erysipelotrichia_Erysipelotrichales_Erysipelotrichaceae	0.687647794370 81	0.00713154853651378	0.509675112389299	0.655759928386239	0.611111380910242	0.759353370813044	0.92384345673614	0.0998416795111929	0.866180395450157	0.864967994392914	0.915850920352629	0.937512059639133
