# Supplementary material for: Fecal microbiota in congenital chloride diarrhea and inflammatory bowel disease
Source: PLoS One. 2022 Jun 9;17(6):e0269561. doi: 10.1371/journal.pone.0269561 (PMC9182261; doi:10.1371/journal.pone.0269561)
Supplement: S10 Table — P values for the changes in fecal microbiota between the baseline and the end of the 3-week follow-up (time3). Data are shown for the group with the standard salt substitution (treatment_standard; 0 weeks vs 3 weeks), and for the group attending the butyrate trial before the trial (before_butyrate; 0 weeks vs 3 weeks), and during the trial (after_butyrate; before butyrate vs after 3 weeks administration of butyrate). Shown are only the taxa with adjusted P values (FDR) <0.1 after CovariateTest. p, P value. FDR, adjusted P value after Benjamini-Hochberg correction. (PDF) [file pone.0269561.s020.pdf]

taxon	time3_treatment _standard_p	time3_treatment _before_butyrate_p	time3_treatment _after_butyrate_p	time3_treatment _standard_lFDR	time3_treatment _before_butyrate_FDR	time3_treatment _after_butyrate_FDR
Firmicutes_Bacilli_Lactobacillales	0.017892013575162	0.979235044034332	**0.002798541234306**	0.214704162901943	0.9799123517855	**0.0426777538231665**
Firmicutes_Bacilli_Lactobacillales_Streptococcaceae	0.0171608497809288	0.9799123517855	**0.00270254804534722**	0.214704162901943	0.9799123517855	**0.0426777538231665**
Firmicutes_Bacilli_Lactobacillales_Streptococcaceae_Streptococcus	0.0277459376523485	0.971505371958582	**0.00130187628192604**	0.237822322734416	0.9799123517855	**0.0397072265987441**
Firmicutes_Clostridia_Clostridiales_Lachnospiraceae	0.23257204321195	**0.00197381610102581**	0.20883792614796	0.4651440864239	**0.0592144830307743**	0.849274233001705
Firmicutes_Clostridia_Clostridiales_Lachnospiraceae_Blautia	NA	0.460514647453641	**0.000477386064889363**	NA	0.668825448278193	**0.0291205499582512**
Firmicutes_Negativicutes_Selenomonadales_Acidaminococcaceae_uncultured	0.361648783527481	0.000924807003372744	0.65224243263961	0.638203735636732	0.0554884202023647	0.864581603689061
