# Supplementary material for: Fecal microbiota in congenital chloride diarrhea and inflammatory bowel disease
Source: PLoS One. 2022 Jun 9;17(6):e0269561. doi: 10.1371/journal.pone.0269561 (PMC9182261; doi:10.1371/journal.pone.0269561)
Supplement: S11 Table — No significant differences were detected between the groups. Samples from 15 out of 16 subjects attending the butyrate trial were available before and after the trial. (PDF) [file pone.0269561.s021.pdf]

Clinical parameters	Group 1 (n=15) (no treatment)		Group 2 (n=15) (treatment group at baseline)		Group 3 (n=15) (treatment group after butyrate trial)	
	(n)	Mean ± SD	(n)	Mean ± SD	(n)	Mean ± SD
Age (years)	15	23.8 ± 15	15	31.2 ± 10.4	15	31.2 ± 10.4
Height (cm)	15	153 ± 31.3	15	170 ± 10	15	170 ± 10
Weight (kg)	15	57.1 ± 27.2	15	69.5 ± 15	15	69.5 ± 15
BMI (kg/m^2)	15	22 ± 5.1	15	24 ± 3.4	15	24 ± 3.4
CRP(mg/L)	15	2.9 (2.9, 7.0)	15	2.9 (2.9, 13)	14	2.9 (2.9, 2.9)
Hemoglobin (g/L)	15	140 ± 13.3	15	142 ± 20	15	140 ± 19
Leukocyte count (E9/L)	15	7 ± 1.5	15	6.5 ± 1.5	15	6.6 ± 1.6
Thrombocyte count (E9/L)	15	335 ± 73	15	324 ± 86	15	330 ± 85
vB-pH	15	7.3 ± 0.06	15	7.3 ± 0.04	15	7.4 ± 0.05
vB-BE (mmol/L)	15	2.5 (0.5, 5.0)	15	3.5 (0.9, 5.3)	15	5 (0.8, 6.4)
P-K (mmol/L)	15	3.8 ± 0.7	15	3.8 ± 0.6	15	3.6 ± 0.6
P-Na (mmol/L)	15	138 ± 2.1	15	140 ± 2.5	15	138 ± 3.3
Ca-Ion (mmol/L)	15	1.2 ± 0.03	15	1.2 ± 0.02	15	1.2 ± 0.03
eGFR ($mL/min/1.73m^2$)	9	97 ± 20	13	104 ± 17	13	100 ± 15
P-Cl (mmol/L)	15	101 ± 5.8	15	102 ± 6.2	15	101 ± 7.3
P-Creatinine (µmol/L)	15	59 ± 20	15	71 ± 13	15	69 ± 14
P-Cystatin C (mg/L)	15	0.9 ± 0.2	15	0.9 ± 0.1	14	0.9 ± 0.1
P-Urea (mmol/L)	15	5.4 ± 2.2	15	5.2 ± 1.2	14	5.3 ± 1.7
Bristol stool classification	12	7	14	7	15	7

The data are presented as mean ± SD or median [interquartile range]. Differences between the mean of multiple groups were compared by one-way analysis of variance (ANOVA). BMI, body mass index. CRP, C-reactive protein. vB, venous blood. BE, base excess. eGFR, estimated glomerular filtration rate. U, urine. Bristol stool scale (BSS): type 1–2 indicates constipation, 3–4 normal, and 5–7 diarrhea: all patients reported the BSS of 7.
