# Supplementary material for: Fecal microbiota in congenital chloride diarrhea and inflammatory bowel disease
Source: PLoS One. 2022 Jun 9;17(6):e0269561. doi: 10.1371/journal.pone.0269561 (PMC9182261; doi:10.1371/journal.pone.0269561)
Supplement: S12 Table — Levels of fecal biomarkers in patients with congenital chloride diarrhea (CLD) before and after butyrate treatment. Samples from 15 out of 16 subjects attending the butyrate trial were available before and after the trial. (PDF) [file pone.0269561.s022.pdf]

**Fecal biomarkers**	**Before butyrate**<br>**(0 – 3wk, n=15)**	**After butyrate**<br>**(4 – 6wk, n=15)**
IAP (U/L)	482 [321 – 655]	441 [308 – 620]
IAP (U/L)/mg protein)	392 [311 – 557]	407 [314 – 576]
Protein (mg/mL)	1.06 [0.97 – 1.28]	1.05 [0.84 – 1.23]
Water (%)	94 [92 – 95]	94 [92 – 94]
IgA (μg/mL)	15.4 [1.6 – 46.0]	13.8 [1.5 – 40.4]
IgA (μg/mg protein)	12.1 [1.7 – 42.4]	11.2 [1.4 – 38.0]
IgG (μg/mL)	0.31 [0.09 – 0.69]	0.17 [0.09 – 0.73]
IgG (μg/mg protein)	0.27 [0.08 – 0.73]	0.13 [0.09 – 0.76]
IgM (μg/mL)	6.0 [0.59 – 26.5]	3.5 [0.47 – 20.7]
IgM (μg/mg protein)	4.3 [0.6 – 26.3]	4.5 [0.6 – 21.1]
Calprotectin (μg/g)	9 [6 – 59]	6 [5 – 33]
MG-H1 (μg/mL)	13.1 [9.6 – 20.7]	12.0 [7.4 – 19.1]*
MG-H1 (μg/mg protein)	11.9 [9.3 – 17.2]	13.0 [6.7 – 17.5]

The data are presented as median [interquartile range]. The butyrate treatment was given over the three weeks period. *p<0.02, comparison between before and after butyrate by the Wilcoxon matched-paired signed rank test. IAP, intestine alkaline phosphatase. MG-H1, methylglyoxal-hydro-imidazolone.
